# Coastal vulnerability assessment of the West African coast to flooding and erosion

**DOI:** 10.1038/s41598-023-48612-5

**Published:** 2024-01-09

**Authors:** Olusegun A. Dada, Rafael Almar, Pierre Morand

**Affiliations:** 1 LEGOS (IRD/CNRS/CNES/Toulouse University), Toulouse, France; 2 Department of Marine Science & Technology, Federal University of Technology Akure, Akure, Nigeria; 3 UMI SOURCE (IRD - UVSQ/PARIS SACLAY), Guyancourt, France

**Keywords:** Environmental sciences, Environmental social sciences, Hydrology, Natural hazards, Ocean sciences

## Abstract

Global coastal areas are at risk due to geomorphological issues, climate change-induced sea-level rise, and increasing human population, settlements, and socioeconomic activities. Here, the study examines the vulnerability of the West African (WA) coast using six satellite-derived geophysical variables and two key socioeconomic parameters as indicators of coastal vulnerability index (CVI). These geophysical and socioeconomic variables are integrated to develop a CVI for the WA coast. Then, the regional hotspots of vulnerability with the main indicators that could influence how the WA coast behaves and can be managed are identified. The results indicate that 64, 17 and 19% of WA coastal areas had high to very high CVI, moderate CVI, and low to very low CVI, respectively. The study reveals that while geophysical variables contribute to coastal vulnerability in WA, socioeconomic factors, particularly high population growth and unsustainable human development at the coast, play a considerably larger role. Some sections of the WA coast are more vulnerable and exposed than others, particularly those in the region's northwestern and Gulf of Guinea regions. Climate change and human presence may amplify the vulnerability in these vulnerable areas in the future. Hence, future coastal economic development plans should be based on a deep understanding of local natural conditions, resource status, and geophysical parameters to prevent negative coastal ecosystem transformation. It is also essential to establish a coastal management plan that would facilitate the development of desired actions and stimulate sustainable management of West African coastal areas.

## Introduction

Coasts are dynamic, complex systems responding to extreme weather events, influenced by the human population, settlements, and socioeconomic activities^1^ growing more rapidly than the global average^2^. The flat morphology of the coastal zone influences its colonization, making it conducive to community development and agricultural expansion. The coast's strategic location near water facilitates easier access to fisheries, raw material transportation, and commerce. The promotion of industries, transportation, tourism, and fishing has greatly improved the economics of coastal cities, resulting in greater population growth and infrastructure development^3,4^. The growing population and economic development on coasts are causing significant environmental changes, resource demands, and exposure to coastal hazards like erosion, flooding as well and salinity intrusion^3–5^. Climate change is worsening these ongoing challenges and its potential implications are causing much concern around the world's coasts^6^.

Being transition areas between both constructive and destructive processes of land and oceans, coastal zones hold significant complexity and high physical mobility^7^. Thus, they can be considered naturally unstable systems whose dynamic balance status can be rapidly altered in the face of climate change. In addition, sea level rise (slow-onset hazard) often leads to coastal disturbances due to catastrophic events, causing erosion and sediment redistribution (rapid-onset hazard^8^). Sometimes, due to climatic and oceanic dynamics, low-lying coastal areas can experience flooding due to an unusually high sea level setup. Higher concentrations of people living along the coast lead to eventual increases in morphological instability in these places and a corresponding intensification of natural processes^9,10^. Coastal populations are increasingly concerned about the impacts of climate change, particularly due to the increasing frequency of extreme events and the Intergovernmental Panel on Climate Change's (IPCC) Sixth Assessment Report^11^.

In West Africa, 31% of the population and the main infrastructures are concentrated in the coastal zone^12^. The West African coast faces increased vulnerability and risk due to natural events like sea-level rise and storm intensities, exacerbated by the region's low-lying status^13^. Moreover, most WA coastal countries are undergoing rapid population growth, urbanization, coastward migration, associated socio-economic growth, and dramatic coastal change^14^. Converging crises: rising seas, fast-growing populations, land pressure, and a lack of low-cost housing face most of WA's coastal cities. The increasing population of coastal communities is posing a threat to natural barriers and ecosystems, exposing them to storm surges and flooding. Thus, to mitigate the effects of rising seas, it will be necessary to simultaneously address multiple causes of coastal hazards. However, no multi-hazard study has assessed the vulnerability index status of the entire WA coast, considering bio-geophysical factors like natural geomorphology, historical shoreline change, coastal slope, wave, tidal range, and sea level rise^15,16^. Therefore, the present study aims to create a comprehensive coastal vulnerability index and identify the population exposed on the WA coast. The results are expected to enhance understanding of WA's coastal vulnerability, enabling stakeholders to anticipate potential impacts and prioritize management efforts to minimize risks.

### The study area settings and present status

**Figure 1:**
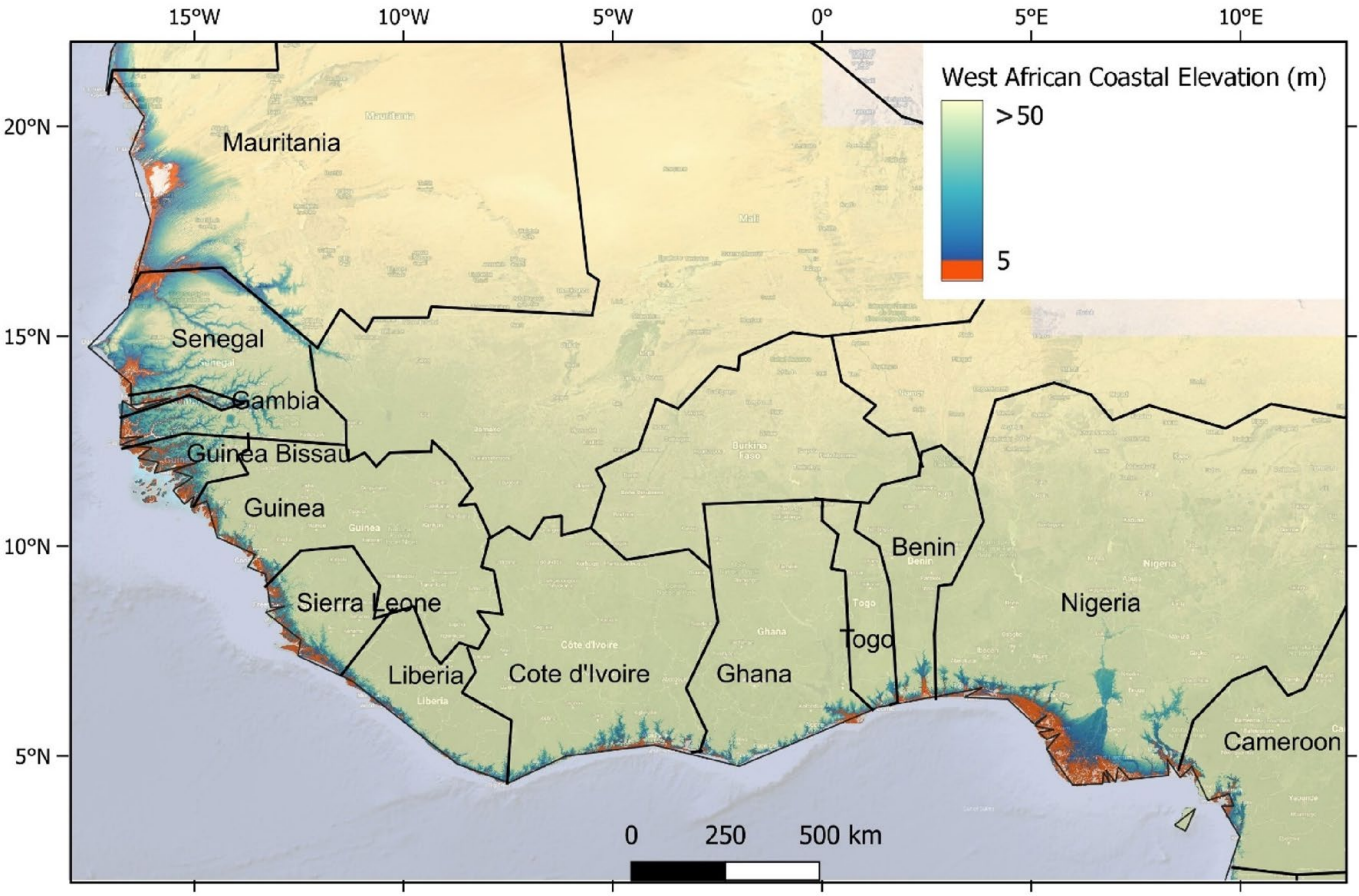
West African coastal elevation (m). Coastal elevation below 5 m is in red (Data source: MERIT DEM). The map in Fig. 1 is generated using data acquired from MERIT DEM (http://hydro.iis.u-tokyo.ac.jp/~yamadai/MERIT_DEM/) in QGIS v.3.24.0 environment (https://www.qgis.org/).

The study area, covering the mainland West African (WA) coastal countries from Mauritania to Nigeria, and Cameroon (Fig. 1), presents a unique coastal geomorphic variability. The coast of West Africa is home to a diverse range of ecosystems and habitats. The biodiversity in this area is influenced by the abundance of estuaries, deltas, coastal lagoons, and nutrient-rich cold-water upwelling. These provide essential habitats for migratory birds, sea turtles, and other ecosystems. Presently, socio-economic activities are increasingly affecting the coastal and marine environment of WA^17–30^.

The long-term trend of people migrating to coastal areas, particularly coastal cities, poses a significant challenge to managing coastal resources. The region is experiencing a rising trend of overexploitation of coastal resources and ecosystems. Due to growing population pressures and a lack of alternative resources to support populations, resource exploitation is becoming unsustainable. Critical coastal ecosystems are damaged or destroyed along coastlines to make room for urban growth, agriculture, aquaculture, port and harbour development, and hydropower dams^31^.

Rapid urbanization in West Africa is a significant concern due to its increasing pressure on the ecosystem and its resources. For instance, the Gambia, Ghana, Mauritania, Liberia, Nigeria, and Cote d'Ivoire have urbanization levels of 50–60%, with a potential global peak of 70% by 2050^32,33^. Besides, sea-level rise and changes in the frequency and power of extreme meteorological events are increasing the impact on coastal flooding and erosion by the acceleration of land loss^34–39^. Sea level rise and increasing extreme events will significantly impact the development of WA coastal areas^32^.

## Results and Discussion

### Results

**Figure 2:**
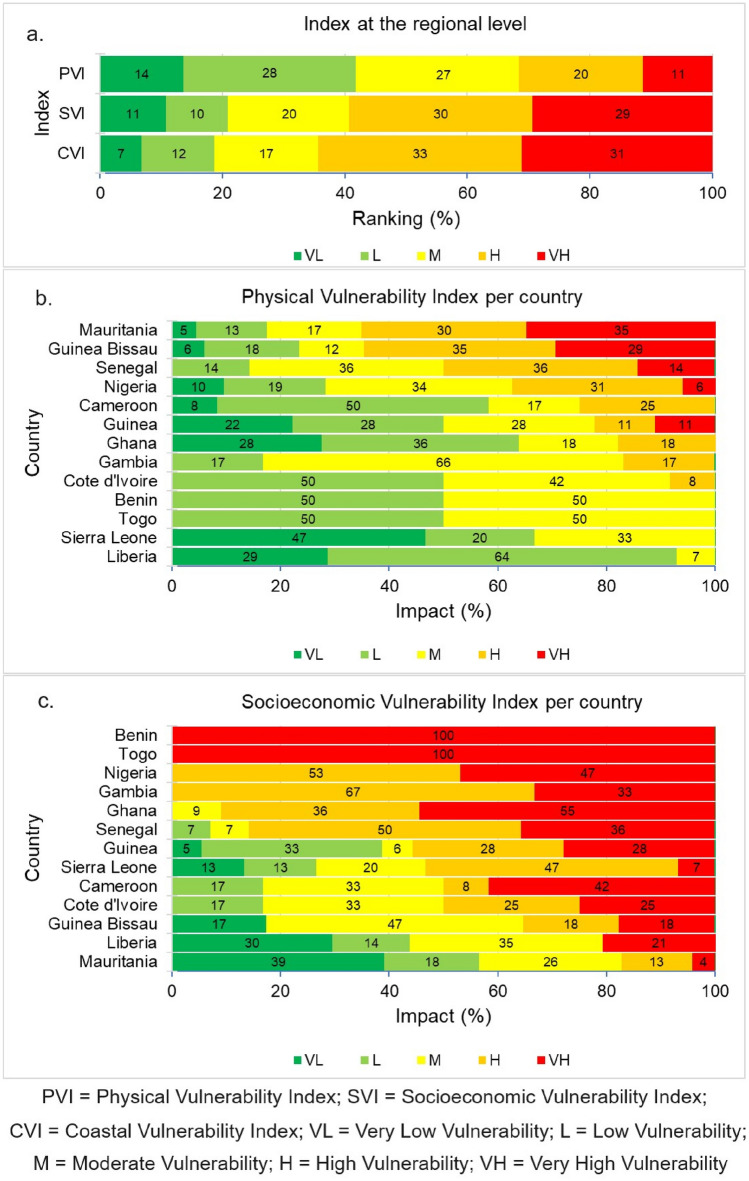
West African coastal vulnerability assessment based on CVI, PVI, and SVI.

The vulnerability of the West African coast is assessed based on three components: PVI, SVI and CVI (Fig. 2).

### Physical vulnerability index (PVI)

**Figure 3:**
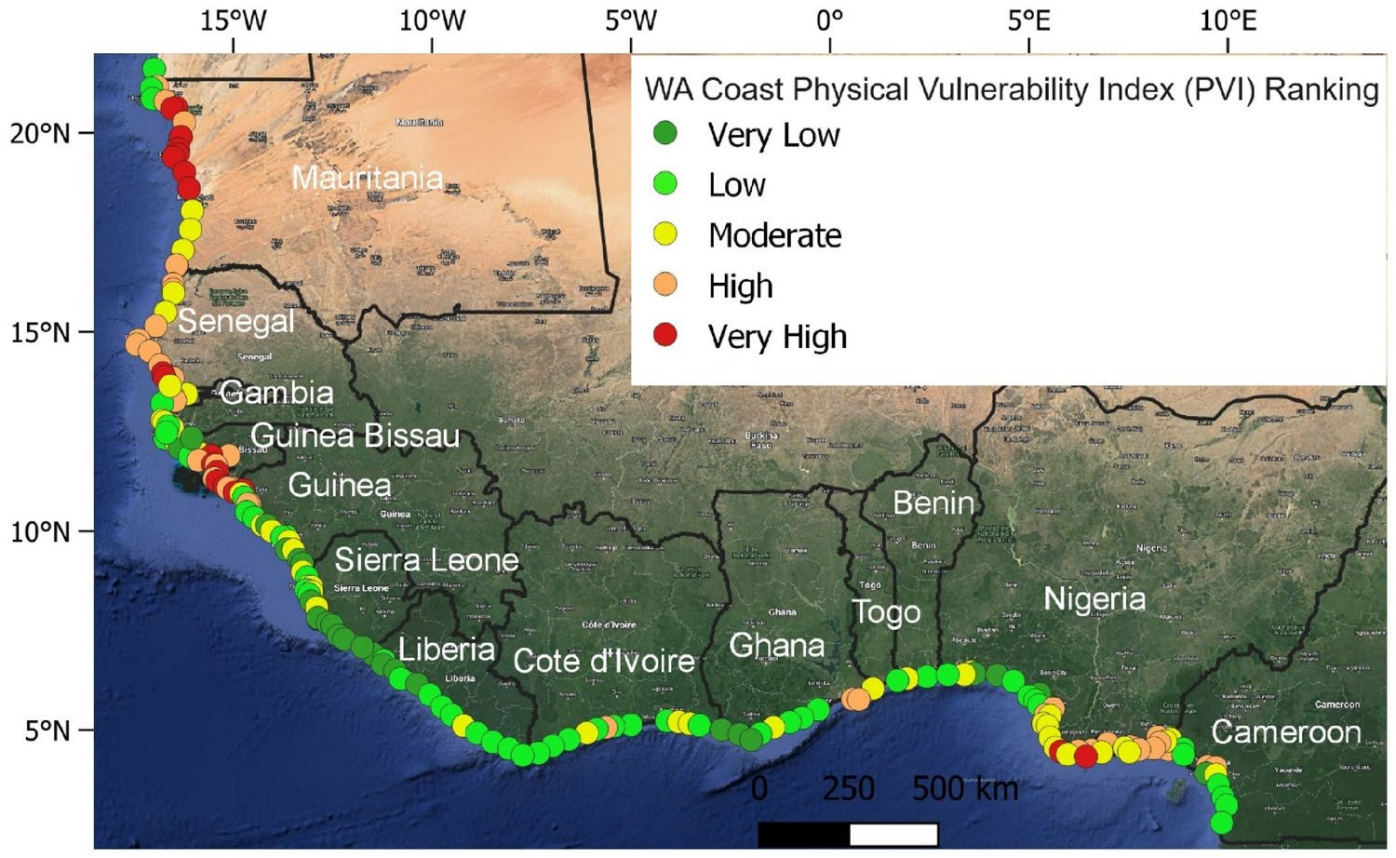
West African physical vulnerability index (PVI). (The map image used in producing the figure was generated using the Google Satellite Hybrid plugin in QGIS v.3.24.0 environment, https://www.qgis.org/).

**Figure 4:**
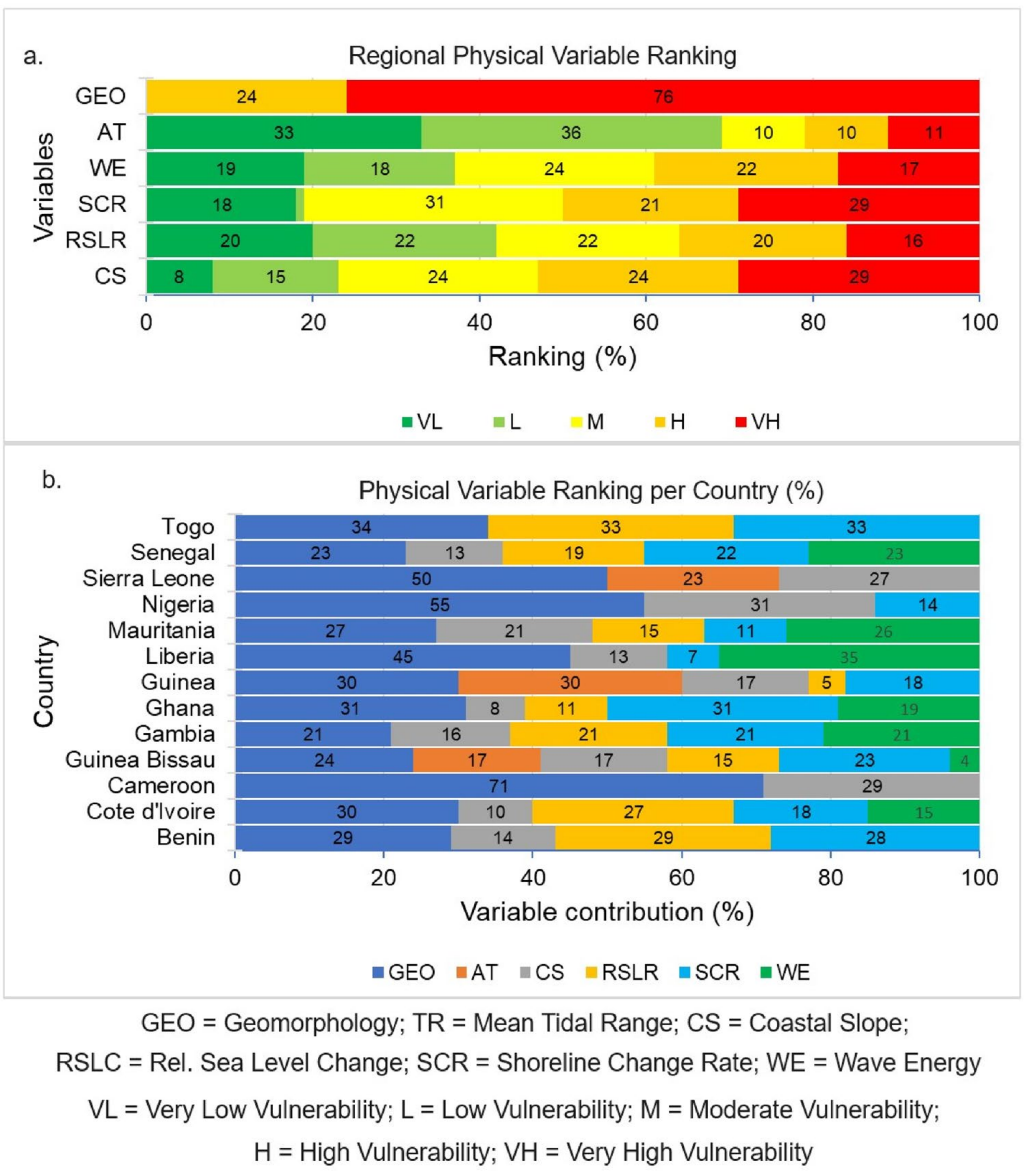
Ranking (%) of various geophysical variables at (**a**) regional level; and (**b**) country level.

There are significant variations in PVIs along the WA coast. On average, the entire WA coast falls under the moderate PVI category. About 31% of the study sites are associated with high to very high vulnerability related to physical processes (PVI) while 27% and 42% of the sites reflected moderate and low to very low PVI (Fig. 2a). As shown in Figs. 2b and 3, the high to very high PVI were mostly found along the coasts of northwestern Senegal (50%), southeastern Nigeria (37%), northern Mauritania (65%), and southeastern Guinea Bissau (64%). Moderate PVIs are found along the coasts of Togo (50%), southeastern Senegal (36%), southeastern Sierra Leone (33%), southwestern Nigeria (34%), northwestern Guinea (29%), Gambia (66%), western Cote d'Ivoire (42%), and Benin (50%). While low to very PVIs are found along the coasts of Togo (50%), southeastern Sierra Leone (67%), Liberia (93%), Guinea (50%), western Ghana (64%), southern Cameroon (58%), western Cote d'Ivoire (50%) and Benin (50%). Geomorphology, coastal slope, and wave energy are all important variables that influence the PVI in the study area (Fig. 4).

### Socioeconomic vulnerability (SVI)

**Figure 5:**
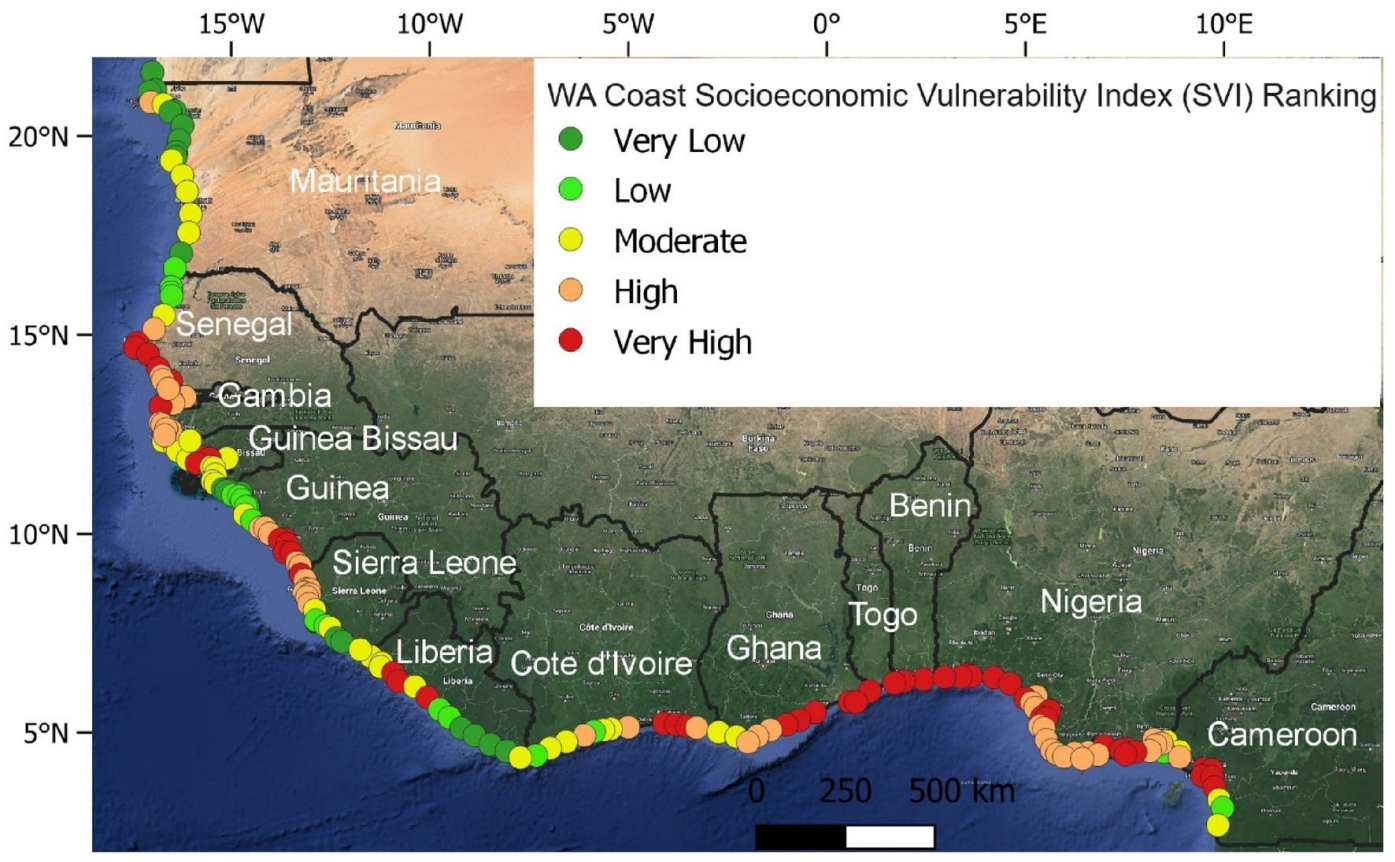
West African socioeconomic vulnerability index (SVI). (The map image used in producing the figure was generated using the Google Satellite Hybrid plugin in QGIS v.3.24.0 environment, https://www.qgis.org/).

About 21% of the study sites are located in areas with very low to low SVI (Fig. 2a). These sites (Figs. 2c, 5) can be found in northern Mauritania (57%), southeastern Sierra Leone (26%), southeastern Liberia (44%), and northwestern Guinea (38%). Moderately SVI locations account for 20% of the study sites (Fig. 2a). These locations (Figs. 2c, 5) can be found along the coasts of Togo (50%), southern Mauritania (26%), northwestern Liberia (35%), northern and southern flanks of Guinea Bissau (47%), and southern Cameroon and western Cote d'Ivoire (33%). Fifty-nine percent of the study sites have high to very high SVI (Fig. 2a) and are located along the coasts of Togo (50%), central and southern Senegal (86%), northwestern Sierra Leone (54%), Nigeria (100%), southeastern Guinea (56%), central and eastern Ghana (91%), Gambia (100%), central Guinea Bissau (36%), northwestern Cameroon and western Cote d'Ivoire (50%), and Benin (100%) (Figs. 2c, 5).

### Overall coastal vulnerability index (CVI)

**Figure 6:**
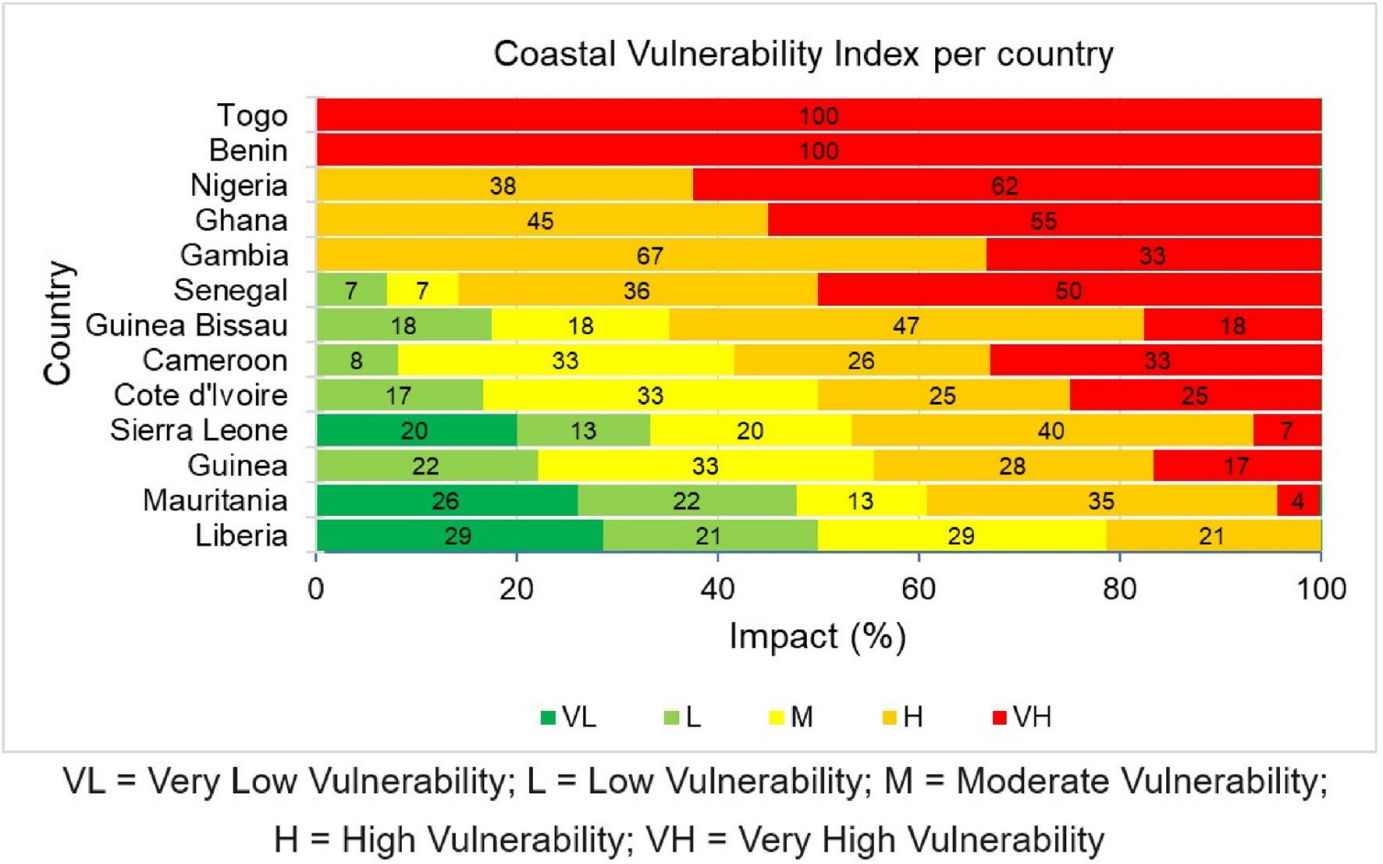
West African coastal vulnerability index per country.

**Figure 7:**
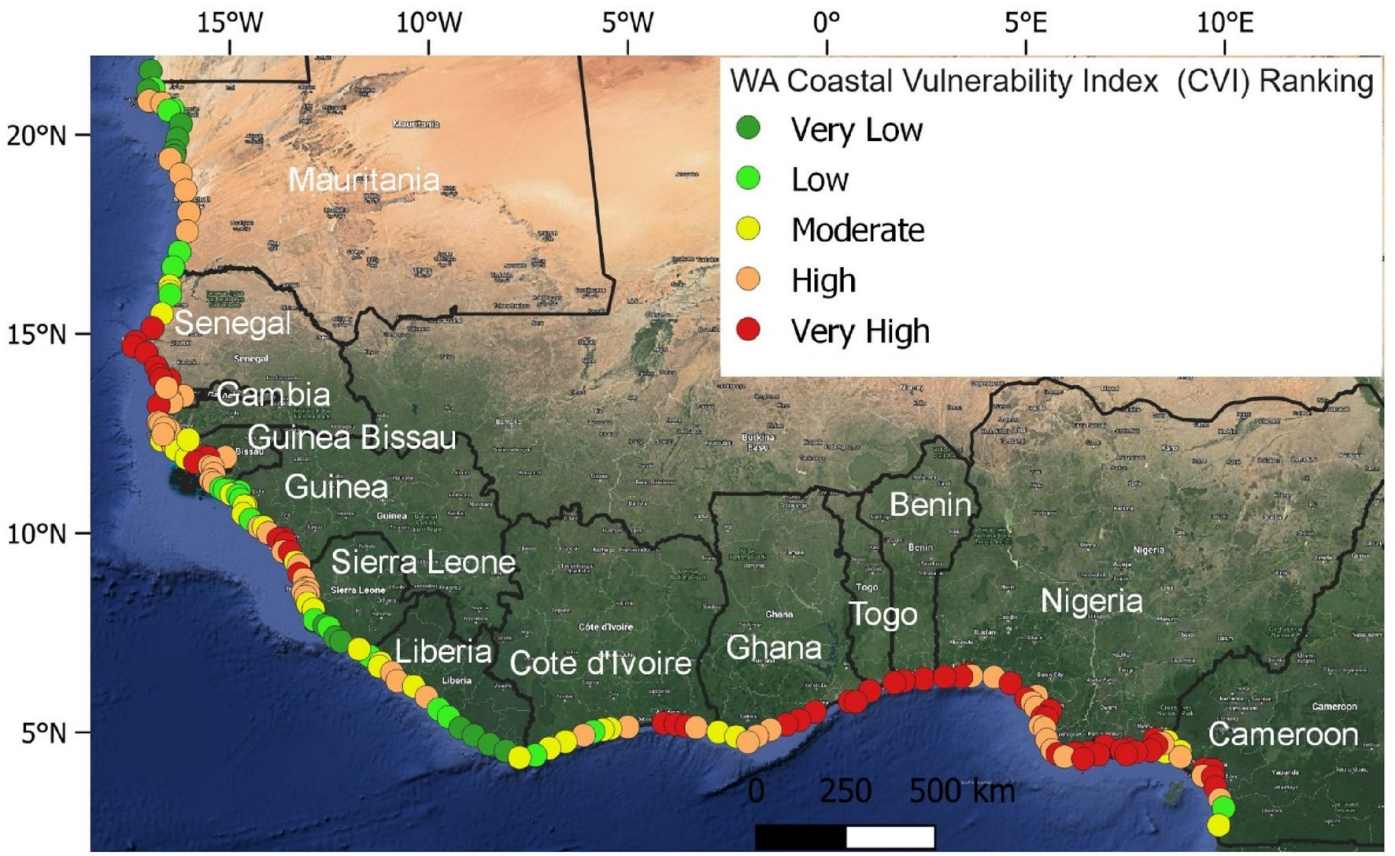
West African overall coastal vulnerability index (CVI). (The map image used in producing the figure was generated using the Google Satellite Hybrid plugin in QGIS v.3.24.0 environment, https://www.qgis.org/).

The CVI was calculated by integrating the PVI and SVI using Eq. 3. About 21% of the study sites fall under the very low to low CVI. As shown in Figs. 6 and 7, they are mainly located in the coastal areas of northern and southern Mauritania (48%), southern Sierra Leone (36%), southeastern Liberia (50%), and Guinea (22%). Moderately CVI occupy 33% of the study sites and they are mainly found along the coasts of northwestern Sierra Leone (20%), northwestern Liberia (29%), Cameroon, Cote d'Ivoire and northern Guinea (33%). High to very high CVI occupy 64% of the study sites and they are mainly situated along the coasts of Togo (100%), central and southern Senegal (86%), northern Sierra Leone (47%), Nigeria (100%), southern Mauritania (39%), central Liberia (21%), southern Guinea (45%), Ghana (100%), Gambia (100%), Guinea Bissau (65%), northwestern Cameroon (59%), eastern Cote d'Ivoire (50%) and Benin (100%) (Figs. 6, 7).

### West Africa vulnerable locations

A more detailed description of the locations that are vulnerable to coastal erosion and flooding hazards along the WA coast is given.

#### Very vulnerable zones

Based on the CVI, some areas are very vulnerable, and most of these areas are located along the northern section of the WA coast, from Mauritania to Senegal, in the western section of WA, from Guinea Bissau to Liberia and in the Gulf of Guinea Coast, from Cote d’Ivoire to Cameroon. As shown in Fig. 7, the central part of Mauritania and central and southern flanks of Senegal coast, the Gambia coast, the southern part of Guinea Bissau and southern Guinea to the northern coast of Sierra Leone displayed a high to very high vulnerability. Other areas along the WA coast with a similar attribute are a location in the eastern part of Cote d’Ivoire, from central Ghana towards the northern Cameroonian coast. Most of these locations have long uninterrupted sand beaches and an unfavourable combination of coastal geomorphology, slope, and erosion trend, mostly under the influence of energetic wave actions and possibly relative sea level rise^40^ (Fig. 4; Supplementary Figs. 1–6).

High to very high SVI are found in the central and southern parts of Senegal, the central part of Guinea Bissau, from the central Guinea coast to the northern part of the Sierra Leone coast. Also, at some locations in central Liberia and the eastern coast of Cote d’Ivoire towards the boundary with Ghana. Then, it extends from the central Ghana coast to the northern part of the Cameroon coast (Fig. 5).

Surprisingly, these high to very high CVI areas (Fig. 7) are equally areas of high to very SVI (Fig. 5). This implies socioeconomic factors have a greater influence on the coastal vulnerability along the WA coast. In addition, these areas are known to be historically vulnerable to coastal flooding^13,28,41–45^ and erosion^22–26,46–48^, thus validating the credibility of this study.

#### Moderately vulnerable zones

Based on CVI, the coasts of northern Senegal, northern Guinea Bissau, Central Guinea, Central and southern Sierra Leone, northern Liberia, western Cote d’Ivoire, and southern Cameroon are found to have a moderate degree of vulnerability (Fig. 7) owing to physical factors such as coastal geomorphology, slope, wave energy, and relative sea level rise (Fig. 4). Based on SVI, the Gambian coast, central Mauritania, northern and southern sections of the Guinea Bissau coast, and the central part of the Guinea Sierra Leone and Cote d’Ivoire coasts, northern Liberia, western Ghana and southern Cameroon fall under moderately vulnerable areas (Fig. 5).

#### Less vulnerable zones

All parts of the WA coastline that have not been mentioned in the previous two categories can be considered less vulnerable. Based on CVI, the northern Mauritania coast, the northern boundary of Senegal, in addition the southern coasts of Sierra Leone and Liberia are the least vulnerable stretches of coastline in WA (Figs. 7). The low vulnerability of these coasts could be because they are low-energy coasts with flat and wide beaches backed by coastal lagoons^49^. Based on SVI, southern Liberia and northern Mauritania coasts have low to very low socioeconomic-related vulnerability (Fig. 5).

### The main vulnerability indicator

**Figure 8:**
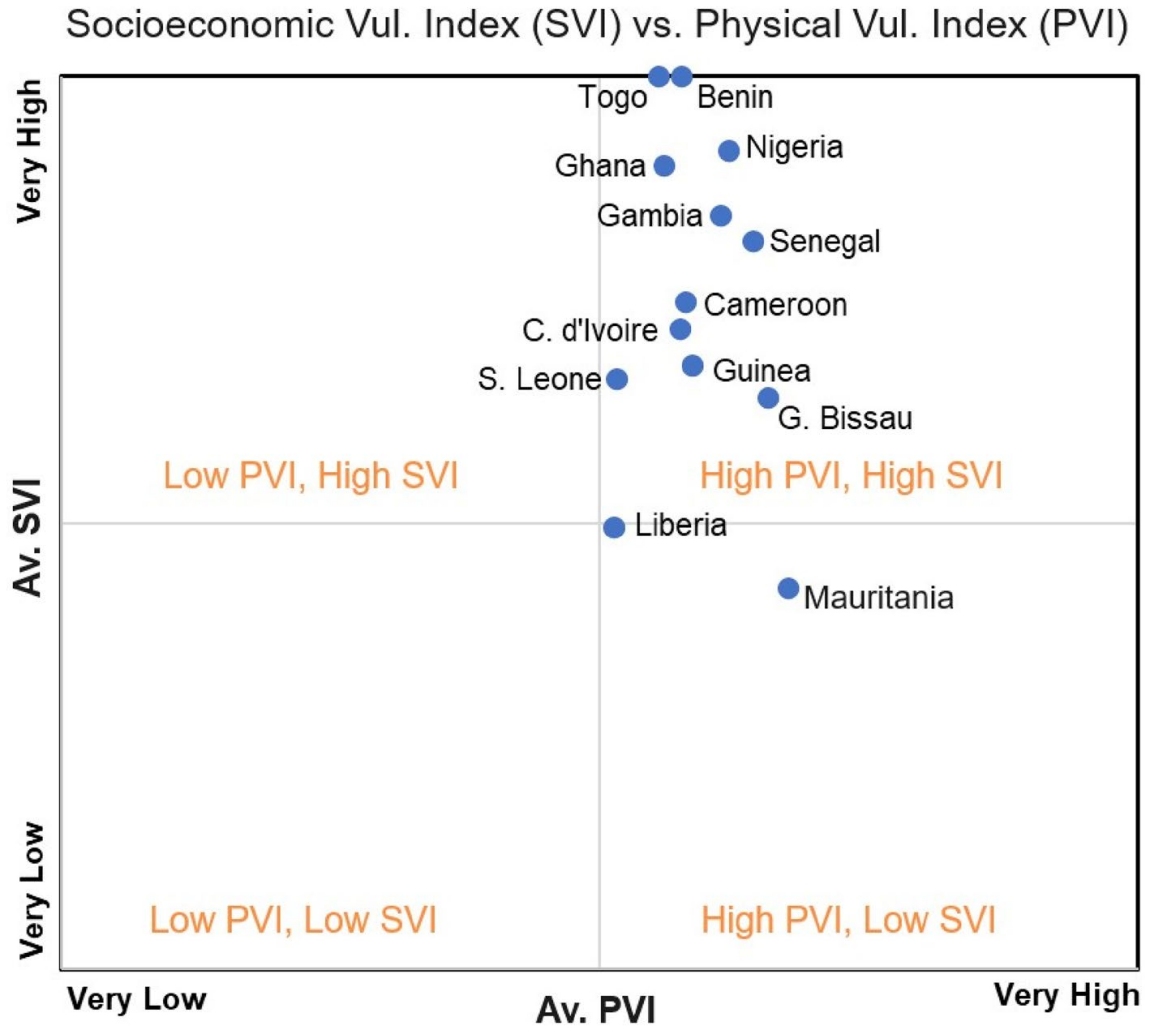
Graphical illustration of PVI and SVI. It shows that the increasing socioeconomic development along the West African coast is a major factor in the WA coastal vulnerability.

Comparison between the PVI and SVI allows for a better assessment of the overall levels of vulnerability of the different sites. To assess potential links between the PVI and the SVI, and to obtain a better understanding of both the magnitudes of change and the economic consequences, Table 1 shows the percentage of PVI and SVI for every WA coastal country. Figure 8 represents the coastal countries using graphical quadrants according to four categories: low PVI/low SVI, low PVI/high SVI, high PVI/low SVI and high PVI/high SVI categories. The average PVI and SVI scores gave interesting results (Table 1; Fig. 8). While Mauritania and Guinea Bissau's PVIs are higher than their SVIs, the SVIs for other WA coastal countries are higher than their PVIs. The SVI is much higher than the PVI at study sites in Benin, Ghana, Nigeria, Togo (100%), Gambia (83.3%) and Senegal (86.8%). This can be explained in terms of high population density and human development in terms of population, built environment and urbanization at these sites (Table 1; Fig. 8).

**Table 1:** Average physical vulnerability index (PVI) and socioeconomic vulnerability index (SVI) in %

Country	PVI (%)	SVI (%)			PVI-SVI (%)
		POP	SETT	POP-SETT	
Benin	–	**100**			–
		–	–	100	
Cote d’Ivoire	25	**75**			–
		25	50	25	
Cameroon	25	**58.3**			16.7
		–	54.5	45.5	
Guinea Bissau	**52.9**	47.1			–
		11.8	82.4	5.8	
Gambia	–	**83.3**			16.7
		–	66.7	33.3	
Ghana	–	**100**			–
		27.3	18.2	54.5	
Guinea	44.4	**55.6**			–
		43.8	33.3	27.7	
Liberia	42.9	**57.1**			–
		56.9	42.9	7.2	
Mauritania	**69.6**	30.4			–
		10.5	89.5		
Nigeria	–	**100**			–
		43.8	12.4	43.8	
Sierra Leone	26.7	**66.7**			6.7
		46.7	46.7	6.6	
Senegal	7.1	**86.8**			7.1
		7.1	57.1	35.8	
Togo	–	**100**			–
		–	–	100	

Significant values are in bold.

The identification of the main factor (physical or socio-economic) that governs the vulnerability of the WA coast is important when adaptation measures are considered. As illustrated in Fig. 8, the vulnerability of the WA coast is dominated by socio-economic factors which are greater than 1 (Fig. 8). Further analysis shows that it is dominated by human development activity (Table 1). The degree of vulnerability associated with socioeconomic variables, especially human development activity, compared with physical variables is high for most countries (Table 1; Fig. 8). Hence adaptation solutions should focus more on socioeconomic factors. The graphic representation can be used at different scales to compare coastal areas, which could help decision-makers prioritize limited resources to protect the most vulnerable areas.

## Discussion

### Impacts of physical processes on WA coastal vulnerability

The variability in the CVI to erosion and flooding along the WA coast is influenced by different (geo)physical variables (Fig. 7). Based on our categorization, the physical variables with the highest dominant impacts on the vulnerabilities of the WA coastline are geomorphology, shoreline change rate, coastal slope, wave climate and sea level rise (Fig. 7; Supplementary Figs. 1–6). The high to very-high vulnerability areas are typically areas of a low coastal slope, vulnerable landform types (deltas, sandy, and muddy beaches), high wave energy coastlines, and a high relative sea-level rise (Supplementary Figs. 1–3). While wave energy is the main physical variable controlling the coastal processes along the WA coastline, the influence of tides is more prominent along the Sierra Leone, Guinea, Guinea Bissau, and Cameroon coasts (Fig. 7; Supplementary Figs. 5–6), possibly due to attenuation of waves (wave sheltering) by offshore islands^50,51^.

The coastal processes along the WA coast between southern Guinea and northern Sierra Leone are influenced by wave energy and tidal currents and are exposed to low to moderate energy, long-period swells (Supplementary Figs. 5–6). A previous study revealed that the highest tidal amplitudes (typically 2.8–4.7 m) are recorded in Guinea, Guinea-Bissau, and Sierra Leone^29^. According to previous studies, relative sea level change is also evident at all locations^14^ (Supplementary Fig. 3).

Our current finding is consistent with the previous studies. For instance, Lopes et al.^16^ developed a coastal vulnerability index for the Guinea-Bissau coast, revealing that 52% of the 87 km coastline has high to very high vulnerability, 20% is moderately vulnerable, and 28% is low to very low. Further, Lopes et al.^16^ observed a rate of 8.79 mm/yr sea level rise in Guinea Bissau. This value, however, may be too high because it was derived using a linear regression fit of 12 data points (one every year) at a single tide gauge, with an R^2^ value of 0.023. The muddy Guinea-Guinea Bissau-northern Sierra Leone sector also experiences significant wave dampening and tidal range amplification due to increased continental shelf width caused by geological offsetting from the Guinea and Sierra Leone fracture zones^29,41,52,53^. This is consistent with the current study, which found that in addition to geomorphology, coastal slope, tidal range, and sea level all have a significant impact on defining coastal vulnerabilities (Supplementary Figs. 1–6).

In Mauritania, geomorphology is generally low in several places below sea level, and it is protected by a thin and fragile dune ridge which can be crossed in some places during strong storms. Aside, port facilities and other human activities at the coastline are exacerbating the vulnerability of the Mauritania coastal area^54^ (Supplementary Figs. 7–8). Based on previous studies^41^, the Mauritanian coast is made up of both a mangrove habitat at the mouth of the Senegal River and energetic beaches in the north. Due to port development, Nouakchott is the area most vulnerable to coastal erosion.

Senegal and Gambia's coasts feature diverse ecosystems, including sandy beaches, volcanic rocky outcrops, and large estuary expanses near the mouths of Senegal, Saloum, Gambia, and Casamance Rivers. The low coasts of Senegal and Gambia are particularly affected by the widespread phenomena of coastal erosion^55^. Retrogression of the coastline has long been a problem for this region of the WA coast in the northwest^41^. In consonance with the present study, a recent study on Senegal's coastal vulnerability index reveals that 70% of the coast has high and very high coastal vulnerability values, primarily in densely populated areas^56^. The study found that 29% of Senegal's coastline is at a high flooding vulnerability index, primarily in the central sector of the most populated districts^56^. Human occupation of coastal zones is a weakness for the coastal areas^57^ because human presence constitutes a factor of exposure to socioeconomic activities^58^. According to Oloyede et al.^59^, 59–65% of Nigeria's coastline is at moderate to high risk of sea-level rise, while coastal populations are highly vulnerable to physical, geomorphological, and socioeconomic stressors.

In Cote d’Ivoire, the entire Ivorian coastal zone is classified as having a moderate vulnerability level by Tano et al.^15^, and the vulnerability grows as one moves eastward. The relative vulnerability of the various sections of the Ivorian coast is significantly influenced by wave energy and geomorphology^15^. While Appeaning Addo^13^ classified the Accra sector of Ghana's coast as moderate risk, Boateng et al.^60^ indicated that Ghana's central and eastern coasts are the most vulnerable, with around 50% of Ghana's 540 km the coastline vulnerable. The approaches and the regions covered may have resulted in these two disparate conclusions on the Ghana coast. While Boateng et al.^60^ examined the whole Ghana coast, Appeaning Addo^13^ exclusively examined the Accra section. The assertion by Boateng et al.^60^ is further corroborated by Charuka et al.^61^ and is consistent with the present study.

The coastal strip between the Volta River delta and the far westernmost part of Benin is densely inhabited (e.g., Lomé, Cotonou) and extremely vulnerable due to migration from inland areas and ongoing development could have a severe impact on the region's socioeconomic conditions and natural ecosystems^62^. The Lomé port's presence as well as other human activities on the coastal plain, such as groundwater extraction that causes subsidence that may result in relative sea level rise, are the main causes of the noticeable erosion of huge coastal sectors with maximum retreat rates of the order of 5 m/yr^62^. The decrease in the Volta River's sediment supply caused by the completion of the Akosombo project is one of the key factors impacting the medium- and long-term evolution of the Volta-Togo-Benin coastal corridor of the WA coast. Also, relative sea level fluctuations that consider tectonic and/or isostatic factors may play a role in the process^62^.

### Implications of increasing socioeconomic development on coastal erosion, subsidence and relative sea level rise

**Figure 9:**
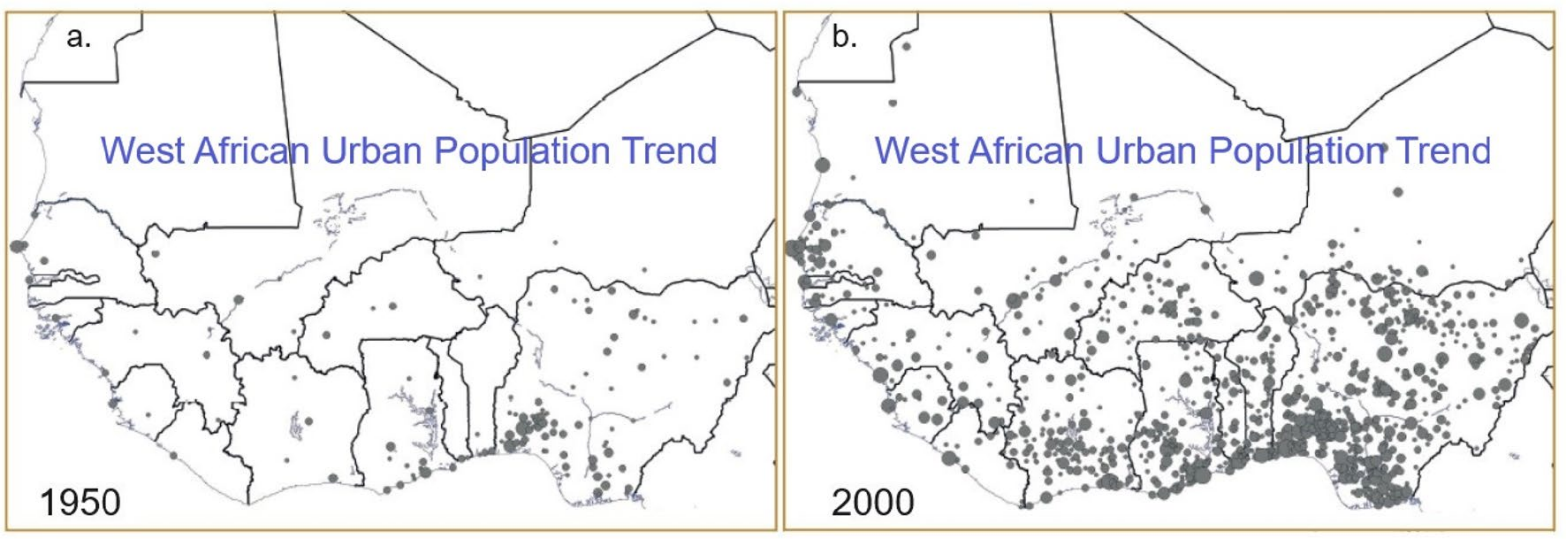
West African urban population trends from 1950 to 2000^72^ (Source: Sahel and West Africa Club Secretariat (SWAC/OECD)). West African urban population trends rose from 125 urban agglomerations and 4 million urban inhabitants in 1950 to 992 urban agglomerations (about a 694% increase) and 78 million urban dwellers (over 1000% increase) in 2000 (31%). The trend continues to grow and is concentrated around coastal towns and cities, especially in the Gulf of Guinea coast.

The WA coast is experiencing a fast-growing population and its attendant development (Fig. 9; Supplementary Figs. 7–8). The average GDP per capita of the Economic Community of West African States (ECOWAS) went from US$2824 in 1990 to US$4373 in 2019, showing a 54.9% growth over this period. The region's GDP per capita in 2019 was 82.6% of Africa's average of $5289^63^. The WA coastal areas house about a third of the region's population and create 56% of its GDP^33^.

With this increased economic growth and accelerated urbanization, the WA coast has seen high-intensity land development and large-scale reclamation in recent decades. This urbanization has had an impact on both the region's coastal towns and cities. It has raised the demand for land, water, and other natural resources; man-made infrastructure and sand mining have contributed to major coastal erosion^33^. The average distance between agglomerations has decreased from 111 to 33 kilometres^64^.

As shown in Fig. 9, WA urban population trends increased from 125 urban agglomerations and 4 million urban inhabitants in 1950–992 urban agglomerations (about 694% increase) and 78 million urban dwellers (over 1000% increase) in 2000, and the trend continues to grow. Seven WA countries, all of which are coastal, have urbanization rates near to or over 40%^64^. Such unsustainable growth is causing the overexploitation of groundwater resources, as well as the reclamation of wetlands or lagoons.

Most crucially, unplanned built-up areas, particularly near coastal cities and towns, pose a threat to the region's socio-ecosystems^27,28,65,66^. Rising urbanization and increasing groundwater overexploitation due to the growing population, particularly in coastal cities, are causing subsiding land, building collapse, and increasing flood risk^65–67^.

The recent co-incidence of subsidence and sea-level rise in coastal cities has garnered greater attention due to the potential for increased future inundation hazards resulting from relative sea level rise^68–71^. The risk of coastal flooding along the WA coast may increase significantly when considering the contribution of sea level rise driven by climate change^28^. Subsidence rates of between − 2 and − 87 mm/yr have been observed in Lagos, Nigeria with the highest rate observed around the coastal zones and areas where heavy structures are built on landfills^67^. With the continued indiscriminate groundwater exploitation, increasing urbanization, and rapid population growth, the subsidence may increase significantly in the future, and this may further escalate the flood rate and other related coastal geohazards^67^. The ongoing discussion is consistent with the findings of the present study. This further lends credence to the fact that socioeconomic development and growth are critical drivers of coastal development and a major contributor to coastal vulnerability along the WA coast (Figs. 2, 8).

### Limitations of the study

Although the approach used in this study offers a helpful assessment of coastal vulnerability at the regional-national scale, the results do contain a significant amount of uncertainty because coarse-resolution data were used. It would therefore be wise to do more thorough, local-scale studies for the places that the present study indicated as being extremely vulnerable, especially if the potential risk to communities or developments is likewise significant in such areas. Apart from population density, the study database is deficient in information on other socioeconomic indicators such as land use. The addition of these extra risk factors may improve the vulnerability rating over the current set of criteria.

### Conclusions and recommendations

This study adopts analysis of remote-sensing data to quantitatively characterize the WA coast's vulnerability to (geo)physical forcing and socioeconomic factors. The study uses six geophysical variables to assess coastal inundation or erosion hazards combined with two key socioeconomic variables to understand the vulnerability of the WA coast, by assigning them a rank ranging from 1 to 5, based on their relative vulnerability factor. Results indicate shows moderate vulnerability on the entire WA coast, with high vulnerability in the northwestern sector (between Mauritania and Guinea Bissau) and the Gulf of Guinea coast (between Cote d’Ivoire and Cameroon). These highly vulnerable areas are linked to the nature of geomorphological landforms, and coastal slopes which resulted in erosion and inundation due to wave heights and sea level rise. This is further complicated by high to very high SVI (population density and human development activities) in the coastal zones of the region. The study illustrates the degree of vulnerable areas which could increase owing to climate change and the impact of increasing human presence.

It is therefore necessary for coastal managers and policymakers in the region to devise the best adaptation strategies using different methods. The development strategy of “coastal sustainability first” should replace “economic development first”. Based on national and regional vulnerabilities, decision-makers, researchers, and coastal stakeholders in the region should consider appropriate adaptation options. Engineering solutions can be incorporated into a portfolio of coastal adaptation strategies. These actions include safeguarding coastal wetlands, stabilizing dunes, replenishing beaches regularly, strengthening and expanding dike systems in specific locations such as harbours and densely urbanized seafronts, enhancing forecasting, warning, and information-dissemination systems, building refugee shelters, and more.

## Methods

This Section presents the procedure used to evaluate the vulnerability due to coastal erosion and flooding along the West African coastal areas.

### The CVI and its components

The overall coastal vulnerability index (CVI) is defined as the combination of the (geo)physical vulnerability index (PVI) and socioeconomic vulnerability index (SVI), using Eq. 2. The PVI and SVI, respectively, examine the physical variables and socioeconomic factors that are responsible for the vulnerability of the WA coast^15,73^ (Supplementary Table 1).

A database comprising physical variables and socioeconomic parameters that strongly represent significant driving processes of coastal erosion and flooding in West Africa was created. The six physical variables are geomorphology (GEO), historical shoreline change rates (SCR), regional coastal slope (CS), relative sea-level change (RSLC), mean wave energy flux (WE) and mean astronomical tidal range (TR). GEO, SCR, and CS are geologic variables that explain shoreline resistance to erosion, long-term erosion/accretion tendency, and susceptibility to flooding. WE, TR, and RSLC are physical process variables that can cause eroded and flooded areas over timescales^74,75^. CS is considered a better parameter than elevation. The present study used population density (POP) and human settlement (SETT, which also represents human developmental activities) data to represent the socioeconomic variables. This is because urbanization of any area is a product of its economic development and population growth which are key drivers for socioeconomic development. SETT data provides combined gridded information on population growth, built-up environments, and urbanization degree^76,77^.

### Data acquisition, ranking and normalization

#### Physical variables

Data on these variables are derived from previous studies, which are based on an analysis of satellite observation and model reanalysis over 23 years between 1993 and 2015, extracted at 204 coastal sites situated along the open coasts of West Africa. The relatively coarse resolution of our dataset is 0.5° (~ 50 km) along the coastline, aiming to capture regional patterns rather than local features that are out of reach and scope here. SCR is extracted from Almar et al.^19^ at the selected study sites for the 1993–2019 period. While CS, RSLC, WE, and TR are derived from Almar et al.^78^. See Almar et al.^78^ for detailed information about these data and how they were acquired. Here, we used the 95-percentile wave local value at each study site. The GEO data is compiled from a collation of previous studies^18,21,27,41,52,79,80^, and these geomorphological characteristics are further observed visually from satellite images and Google Earth.

#### Socioeconomic variables

The socioeconomic datasets, comprising population density (POP) and human settlement (SETT) were obtained from the Center for International Earth Science Information Network (CIESIN), Columbia University at (https://sedac.ciesin.columbia.edu/data/set/gpw-v4-population-density-rev11) and (https://sedac.ciesin.columbia.edu/data/set/ghsl-population-built-up-estimates-degree-urban-smod/data-download). These data are in Geo TIFF format at a spatial resolution of ~ 1 km (30 arc-seconds). The socioeconomic (population density and human development) variables are employed here to determine their contribution to the coastal vulnerability of the WA coast in terms of erosion and floods. Variables indicating housing and infrastructure stability, as well as the existence (or lack thereof) of an emergency plan in the event of flooding, should have been analyzed. However, such data is not available for the entire West African coastline. Although all estimations are simply quantitative approaches that cannot directly reflect physical processes and socioeconomic pressures and their consequences, they can be used to identify vulnerable or risk areas.

#### Ranking and weighting

Depending on the measured values, different systems have different rankings and ranges of variables^81^. Previous studies^75,81–87^ have employed three to five classes of ranking. Following Mendoza et al.^56^, a scale of 1–5 was assigned to each variable (Supplementary Table 1). This was previously used by Koroglu et al.^88^, Thieler and Hammar-Klose^86^ and Gornitz et al.^89^.

This ranking indicates the degree of vulnerability, with 5 contributing the most strongly (very high—VH) vulnerability and 1 contributing the least (very low—VL) vulnerability. The 1–5 scale standardizes the scoring system for each variable as a new no-dimensional variable, allowing for a mathematical combination of measured units (Supplementary Table 1). The classification method based on Natural Breaks is used to identify breakpoints by looking at groups and patterns inherent in the data. This method uses a rather complex statistical formula (Jenks optimization) that minimizes the sum of the variance within each of the classes^90^.

According to Denner et al.^91^, depending on the impact's significance, socioeconomic and physical variables respond in different ways or exert varying degrees of influence. As a result, each variable was given a weight based on its value and the associated perceived risk level. Once each variable has been assigned a five-level value and weighted, the numerical values are summed within the geographic information system (GIS), and the PVI and SVI are computed for each site using Eqs. (1 and 2).

### Coastal Vulnerability Index (CVI) calculations

The CVIs to erosion and flooding were evaluated for 204 sites based on the PVI and SVI (Supplementary Table 1). The PVI was determined by integrating the normalized values of the physical variables using Eq. (1).

1 $${\text{CVI}} = {\text{Sqrt}}\left\{ {\left( {\left( {{\text{a}}_{{1}}^{{2}} + {\text{a}}_{{2}}^{{2}} + {\text{a}}_{{3}}^{{2}} + {\text{a}}_{{4}}^{{2}} + {\text{a}}_{{5}}^{{2}} + {\text{a}}_{{6}}^{{2}} } \right)} \right)/6} \right\}$$

where a_1_ = geomorphology (GEO), a_2_ = shoreline change rate (SCR), a_3_ = coastal slope (CS), a_4_ = relative sea level change (RSLC), a_5_ = wave energy flux (WE), and a_6_ = astronomical tide range (TR). The SVI was determined by integrating the normalized values of the socioeconomic variables using Eq. (2).

2 $${\text{SVI}} = {\text{Sqrt}}\left\{ {\left( {\left( {{\text{b}}_{{1}}^{{2}} + {\text{b}}_{{2}}^{{2}} } \right)/2} \right)} \right\}$$

where b_1_ = population density (POP), b_2_ = human settlement (SETT; comprising population growth, built-up environments, and urbanization degree).

To identify the primary component (physical or socioeconomic) that has a substantial impact on the vulnerability of the corresponding area, the ratio of the normalized SVI to the corresponding normalized CVI was calculated. When the ratio is less than 1, the vulnerability of the corresponding portion is heavily dependent on physical variables. When it equals 1, the coastal section is influenced equally by physical and socioeconomic forces. When the ratio is greater than 1, the socioeconomic variables are the dominant vulnerability factors.

The overall coastal vulnerability index (CVI) is estimated by combining both the PVI and SVI, using Eq. (3):

3 $${\text{CVI}} = \left( {{\text{PVI}} + {\text{SVI}}} \right)/{2}$$

Subsequently, the estimated CVI was classified into five vulnerability classes (very low, low, moderate, high, and very high) based on the Jenks natural classification method^90^ within the GIS. Finally, the results of the analyses are presented as tables and figures.

### Supplementary Information

Supplementary Information.

## Data Availability

The raw data that support the findings of this study are already available online, freely. We used sea level observation from AVISO (https://www.aviso.altimetry.fr/en/data/products/auxiliary-products/dynamic-atmospheric-correction/description-atmospheric-corrections.html), tide from FES2014 tide global atlas^92^, waves from ERA5 (https://cds.climate.copernicus.eu/cdsapp#!/dataset/reanalysis-era5-single-levels). Topography is computed by Almar et al.^47^ from the AW3D30 digital elevation model from JAXA^93,94^. Shoreline mobility (computed in Almar et al.^19^) is derived from 1993 to 2019 using multiple satellite acquisitions provided by Landsat (NASA) missions 5, 7, and 8. The socioeconomic datasets, comprising population density (POP) and human settlement (SETT) are from the Center for International Earth Science Information Network (CIESIN), Columbia University (https://sedac.ciesin.columbia.edu/data/set/gpw-v4-population-density-rev11) and (https://sedac.ciesin.columbia.edu/data/set/ghsl-population-built-up-estimates-degree-urban-smod/data-download).

## References

[1] Balica SF, Wright NG, van der Meulen F (2012). A flood vulnerability index for coastal cities and its use in assessing climate change impacts. Nat. Hazards.

[2] Neumann B, Vafeidis AT, Zimmermann J, Nicholls RJB (2015). Future coastal population growth and exposure to sea-level rise and coastal flooding—A global assessment. PLoS ONE.

[3] Nguyen QH (2021). Impact of investment in tourism infrastructure development on attracting international visitors: A nonlinear panel ARDL approach using Vietnam’s data. Economies.

[4] Stanchev H, Stancheva M, Young R (2015). Implications of population and tourism development growth for Bulgarian coastal zone. J. Coast. Conserv..

[5] Abir, L. M. Impact of tourism in coastal areas: Need of sustainable tourism strategy. Available from http://www.coastalwiki.org/wiki/Impact_of_tourism_in_coastal_areas:_Need_of_sustainable_tourism_strategy (2023).

[6] Nicholls RJ, Hoozemans FMJ (1996). The Mediterranean: Vulnerability to coastal implications of climate change. Ocean Coast. Manag..

[7] Masselink, G. & Gehrels, R. (eds) *Coastal Environments & Global Change* 448 (Wiley-Blackwell, New York, 2014).

[8] Bonetti J, Woodroffe CD, Bartlett D, Celliers L (2017). Spatial analysis techniques and methodological approaches for coastal vulnerability assessment. Geoinformatics for Marine and Coastal Management.

[9] Hummell BML, Cutter SL, Emrich C (2016). Social vulnerability to natural hazards in Brazil. Int. J. Disast. Risk Sci..

[10] Kantamaneni K (2016). Counting the cost of coastal vulnerability. Ocean Coast. Manag..

[11] IPCC. Climate change 2022: Impacts, adaptation, and vulnerability. Contribution of working group II to the sixth assessment report of the intergovernmental panel on climate change (eds Pörtner, H.-O., Roberts, D. C., Tignor, M., Poloczanska, E. S., Mintenbeck, K., Alegría, A., Craig, M., Langsdorf, S., Löschke, S., Möller, V., Okem, A. & Rama, B.) 3056 (Cambridge University Press, Cambridge, UK and New York, NY, USA, 2022).

[12] World Bank Group. A partnership for saving West Africa’s coastal areas. http://pubdocs.worldbank.org/en/622041448394069174/1606426-WACA-Brochure.pdf (2016).

[13] Appeaning Addo K (2013). Assessing coastal vulnerability index to climate change: The case of Accra-Ghana. J. Coast. Res..

[14] Nyadzi E, Bessah E, Kranjac-Berisavljevic G (2021). Taking stock of climate change induced sea level rise across the West African coast. Environ. Claims J..

[15] Tano R, Aman A, Toualy E, Kouadio Y, François-Xavier B, Addo K (2018). Development of an integrated coastal vulnerability index for the Ivorian coast in West Africa. J. Environ. Prot..

[16] Lopes NDR, Li T, Matomela N (2022). Coastal vulnerability assessment based on multi-hazards and bio-geophysical parameters. Case study-northwestern coastline of Guinea-Bissau. Nat. Hazards.

[17] Abessolo GO (2023). African coastal camera network efforts at monitoring ocean, climate, and human impacts. Sci. Rep..

[18] Almar R, Stieglitz T, Addo KA (2022). Coastal zone changes in West Africa: Challenges and opportunities for satellite earth observations. Surv. Geophys..

[19] Almar R, Boucharel J, Graffin M (2023). Influence of El Niño on the variability of global shoreline position. Nat. Commun..

[20] Anthony EJ, Randazzo G, Jackson DW, Cooper JAG (2015). Patterns of sand spit development and their management implications on deltaic, drift-aligned coasts: The cases of the Senegal and Volta River delta spits, West Africa. Sand and Gravel Spits.

[21] Anthony EJ, Almar R, Aagaard T (2016). Recent shoreline changes in the Volta River delta, West Africa: The roles of natural processes and human impacts. Afr. J. Aquat. Sci..

[22] Anthony EJ, Almar R, Besset M, Reyns J, Laibi R, Ranasinghe R, Vacchi M (2019). Response of the Bight of Benin (Gulf of Guinea, West Africa) coastline to anthropogenic and natural forcing, part 2: Sources and patterns of sediment supply, sediment cells, and recent shoreline change. Cont. Shelf Res..

[23] Dada OA, Qiao LL, Ding D, Li GX, Ma YY, Wang LM (2015). Evolutionary trends of the Niger Delta shoreline during the last 100 years: Responses to rainfall and river discharge. Mar. Geol..

[24] Dada OA, Li GX, Qiao LL, Ding D, Ma YY, Xu JS (2016). Seasonal shoreline behaviours along the arcuate Niger Delta coast: Complex interaction between fluvial and marine processes. Cont. Shelf Res..

[25] Dada OA, Li G, Qiao LL (2018). Recent Niger Delta shoreline response to Niger River hydrology: Conflict between force of Nature and Humans. J. Afr. Earth Sci..

[26] Dada OA, Agbaje AO, Adesina RB, Asiwaju-Bello YA (2019). Effect of coastal land use change on coastline dynamics along the Nigerian transgressive Mahin mud coast. J. Ocean Coast. Manag..

[27] Dada O, Almar R, Morand P, Menard F (2021). Towards West African coastal social-ecosystems sustainability: Interdisciplinary approaches. Ocean Coast. Manag..

[28] Dada OA, Almar R, Morand P (2023). Future socioeconomic development along the West African coast forms a larger hazard than sea level rise. Nat. Commun. Earth Environ..

[29] Diop S, Fabres J, Pravettoni R, Diop S, Barusseau JP, Descamps C (2014). The western and central Africa land-sea interface: A vulnerable, threatened, and important coastal zone within a changing environment. The Land/Ocean Interactions in the Coastal Zone of West and Central Africa. Estuaries of the World.

[30] Ly CK (1980). The role of the Akosombo Dam on the Volta River in causing coastal erosion in central and eastern Ghana (West Africa). Mar. Geol..

[31] Diop S, Arthurton R, Scheren P, Wolanski E, McLusky DS (2011). The coastal and marine environment of Eastern and Western Africa: Challenges to sustainable management and socioeconomic development. Treatise on Estuarine and Coastal Science.

[32] OECD. Development at a Glance: Statistics by Region—Africa. 2020. Available online: https://stats.oecd.org/Index.aspx?DataSetCode=Table2A (accessed on 22 October 2023).

[33] Croitoru, L., Miranda, J. J., Sarraf, M. The cost of coastal zone degradation in West Africa, World Bank Group Report. 2019. Available online: https://openknowledge.worldbank.org/bitstream/handle/10986/31428/135269-Cost-of-Coastal-Degradation-in-West-Africa-March-2019.pdf?sequence=1 (accessed on 22 October 2023).

[34] World Bank. Effects of climate change on coastal erosion and flooding in Benin, Côte d'Ivoire, Mauritania, Senegal, and Togo. World Bank Technical Report, 127 (2020).

[35] Marti, F., Cazenave, A., Birol, F., Marcello Passaro, P., Fabien Le ´ger, F., Nin˜o, F., Almar, R., Benveniste, J. & Legeais, J. F. Altimetry-based sea level trends along the coasts of Western Africa. *Adv. Space Res.* (2019).

[36] Appeaning AK (2015). Monitoring sea level rise-induced hazards along the coast of Accra in Ghana. Nat. Hazards.

[37] Appeaning Addo K, Larbi L, Amisigo B, Ofori-Danson PK (2011). Impacts of coastal inundation due to climate change in a cluster of urban coastal communities in Ghana, West Africa. Remote Sens..

[38] Dada OA, Almar R, Oladapo MI (2020). Recent coastal sea-level variations and flooding events in the Nigerian Transgressive Mud coast of Gulf of Guinea. J. Afr. Earth Sci..

[39] Failler P, Touron-Gardic G, Drakeford B, Sadio O, Traoré M-S (2020). Perception of threats and related management measures: The case of 32 marine protected areas in West Africa. Mar. Policy.

[40] Almar R, Kestenare E, Boucharel J (2019). On the key influence of remote climate variability from Tropical Cyclones, North and South Atlantic mid-latitude storms on the Senegalese coast (West Africa). Environ. Res. Commun..

[41] Alves B, Angnuureng DB, Morand P, Almar R (2020). A review on coastal erosion and flooding risks and best management practices in West Africa: What has been done and should be done. J. Coast. Conserv..

[42] Angnuureng DB, Addo KA, Almar R, Dieng H (2018). Influence of sea level variability on a micro-tidal beach. Nat. Hazards.

[43] Brown, S., Kebede, A. S. & Nicholls, R. J. Sea-level rise and impacts in Africa, 2000–2100 (2011) https://research.fit.edu/media/site-specific/researchfitedu/coast-climate-adaptation-library/africa/regional---africa/Brown-et-al.--2009.--SLR--Impact-in-Africa.pdf (Accessed online on 08 November 2023).

[44] Tano RA, Aman A, Kouadio KY, Toualy E, Ali KE, Assamoi P (2016). Assessment of the Ivorian coastal vulnerability. J. Coast. Res..

[45] Vousdoukas MI, Clarke J, Ranasinghe R, Reimann L, Khalaf N, Duong TM, Ouweneel B, Sabour S, Iles CE, Trisos CH, Feyen L, Mentaschi L, Simpson NP (2022). African heritage sites threatened as sea-level rise accelerates. Nat. Clim. Change.

[46] Abessolo OG, Hoan LX, Larson M, Almar R (2021). Modeling the Bight of Benin (Gulf of Guinea, West Africa) coastline response to natural and anthropogenic forcing. Reg. Stud. Mar. Sci..

[47] Almar R, Kestenare E, Reyns J, Jouanno J, Anthony EJ, Laibi R, Ranasinghe R (2015). Response of the Bight of Benin (Gulf of Guinea, West Africa) coastline to anthropogenic and natural forcing, Part1: Wave climate variability and impacts on the longshore sediment transport. Cont. Shelf Res..

[48] Dada OA, Li GX, Qiao LL, Ding D, Ma YY, Xu JS, Li P, Yang J (2016). Response of wave and coastline evolution to global climate change off the Niger Delta during the past 110 years. Mar. Syst..

[49] Aman A (2019). Physical forcing induced coastal vulnerability along the Gulf of Guinea. J. Environ. Prot..

[50] de Ponce León S, Guedes Soares C (2010). The sheltering effect of the Balearic Islands in the hindcast wave field. Ocean Eng..

[51] Soares CG (2005). On the sheltering effect of islands in ocean wave models. J. Geophys. Res. Oceans.

[52] Anthony EJ (2006). The muddy tropical coast of West Africa from Sierra Leone to Guinea-Bissau: Geological heritage, geomorphology and sediment dynamics. Afr. Geosci. Rev..

[53] Anthony EJ (1995). Beach-ridge development and sediment supply: Examples from West Africa. Mar. Geol..

[54] Niang AJ, Al Saud MM (2022). Remote sensing and GIS application for natural hazards assessment of the Mauritanian coastal zone. Applications of Space Techniques on the Natural Hazards in the MENA Region.

[55] Thior M, Sané T, Dièye E-HB, Sy O, Cissokho D, Ba BD, Descroix L (2019). Coastline dynamics of the northern lower Casamance (Senegal) and southern Gambia littoral from 1968 to 2017. J. Afr. Earth Sci..

[56] Mendoza E, Salameh E, Sakho I, Turki I, Almar R, Ojeda E, Deloffre J, Frappart F, Laignel B (2023). Coastal flood vulnerability assessment, a satellite remote sensing and modeling approach. Remote Sens. Appl. Soc. Environ..

[57] Meur-Férec C, Deboudt P, Morel V (2008). Coastal risks in France: An integrated method for evaluating vulnerability. J. Coast. Res..

[58] Mclaughlin S, Cooper JAG (2010). A multi-scale coastal vulnerability index: A tool for coastal managers?. Environ. Hazards.

[59] Oloyede MO, Williams AB, Ode GO, Benson NU (2022). Coastal vulnerability assessment: A case study of the Nigerian coastline. Sustainability.

[60] Boateng I, Wiafe G, Jayson-Quashigah P-N (2017). Mapping vulnerability and risk of Ghana’s coastline to sea level rise. Mar. Geodesy.

[61] Charuka B, Angnuureng DB, Brempong EK, Agblorti SK, Antwi Agyakwa KT (2023). Assessment of the integrated coastal vulnerability index of Ghana toward future coastal infrastructure investment plans. Ocean Coast. Manag..

[62] Guerrera F, Tramontana M, Nimon B, Essotina Kpémoua K (2021). Shoreline changes and coastal erosion: The case study of the coast of Togo (Bight of Benin, West Africa Margin). Geosciences.

[63] Aikins, E. R. ECOWAS/West Africa. Published online at futures.issafrica.org. Retrieved from https://futures.issafrica.org/geographic/regions/ecowas/ (2023) [Accessed online 08 November 2023].

[64] Hitimana, L., Heinrigs, P. & Tremolieres, M. West African urbanisation trends. West African Futures 01 (2011). https://www.oecd.org/swac/publications/48231121.pdf

[65] Cian F, Blasco JMD, Carrera L (2019). Sentinel-1 for monitoring land subsidence of coastal cities in Africa using PSInSAR: A methodology based on the integration of SNAP and StaMPS. Geosciences.

[66] Ohenhen LO, Shirzaei M (2022). Land subsidence hazard and building collapse risk in the coastal city of Lagos, West Africa. Earth's Future.

[67] Ikuemonisan FE, Ozebo VC (2020). Characterisation and mapping of land subsidence based on geodetic observations in Lagos, Nigeria. Geodesy Geodyn..

[68] Nicholls RJ, Lincke D, Hinkel J, Brown S, Vafeidis AT, Meyssignac B, Fang J (2021). A global analysis of subsidence, relative sea-level change and coastal flood exposure. Nat. Clim. Change.

[69] Restrepo-Ángel JD, Mora-Páez H, Díaz F, Govorcin M, Wdowinski S, Giraldo-Londoño L (2021). Coastal subsidence increases vulnerability to sea level rise over twenty first century in Cartagena, Caribbean Colombia. Sci. Rep..

[70] Shirzaei M, Freymueller J, Törnqvist TE, Galloway DL, Dura T, Minderhoud PS (2021). Measuring, modelling and projecting coastal land subsidence. Nat. Rev. Earth Environ..

[71] Wu T, Barrett J (2022). Coastal land use management methodologies under pressure from climate change and population growth. Environ. Manag..

[72] Sahel and West Africa Club Secretariat (SWAC/OECD). In: Hitimana, L., Heinrigs, P., and Tremolieres, M. West African urbanisation trends. West African Futures 01. https://www.oecd.org/swac/publications/48231121.pdf (2011)

[73] Kantamanenia K, Phillip M, Thomas T, Jenkins R (2018). Assessing coastal vulnerability: Development of a combined physical and economic index. Ocean Coast. Manag..

[74] Pendleton, E. A., Thieler, E. R., Williams, S. J. & Beavers, R. S. Coastal vulnerability assessment of Padre Island National Seashore (PAIS) to Sea-level rise. USGS report No 2004-1090 (2004). Available from: http://pubs.usgs.gov/of/2004/1090/a.

[75] Pendleton, E. A., Barras, J. A., Williams, S. J. & Twichell, D. C. Coastal vulnerability assessment of the Northern Gulf of Mexico to sea-level rise and coastal change: U.S. geological survey open-file report 2010-1146. http://pubs.usgs.gov/of/2010/1146/ (2010).

[76] Joint Research Centre - JRC - European Commission and Center for International Earth Science Information Network - CIESIN - Columbia University. Global Human Settlement Layer: Population and Built-Up Estimates, and Degree of Urbanization Settlement Model Grid. Palisades, New York: NASA Socioeconomic Data and Applications Center (SEDAC). 10.7927/h4154f0w (2021). (Accessed online on 08 November 2023).

[77] Florczyk A. J., Corbane, C., Ehrlich, D., Freire, S., Kemper, T., Maffenini, L., Melchiorri, M., Pesaresi, M., Politis, P., Schiavina, M., Sabo, F. & Zanchetta, L. GHSL data package 2019, EUR 29788 EN. ISBN 978-92-76-13186-1, JRC 117104. Luxembourg: Publications Office of the European Union. 10.2760/290498 (2019).

[78] Almar R, Ranasinghe R, Bergsma EWJ, Diaz H, Melet A, Papa F, Vousdoukas M, Athanasiou P, Dada O, Almeida LP, Kestenare E (2021). A global analysis of extreme coastal water levels with implications for potential coastal overtopping. Nat. Commun..

[79] Hayden B, Vincent M, Resio D, Biscoe C, Dolan R (1973). Classification of the Coastal Environments of the World: Part II—Africa.

[80] Allersma E, Tilmans WM (1993). Coastal conditions in West Africa—A review. Ocean Coast. Manag..

[81] Musa ZN, Popescu I, Mynett A (2014). The Niger Delta's vulnerability to river floods due to sea level rise. Nat. Hazards Earth Syst. Sci..

[82] El-Shahat S, El-Zafarany AM, El Seoud TA (2021). Vulnerability assessment of African coasts to sea level rise using GIS and remote sensing. Environ. Dev. Sustain..

[83] Kumar T, Kunte P (2012). Coastal vulnerability assessment for Chennai, east coast of india using geospatial techniques. J. Nat. Hazards.

[84] Yin J, Yin Z, Wang J, Xu S (2012). National assessment of coastal vulnerability to sea-level rise for the Chinese coast. J. Coast. Conserv..

[85] Dinh Q, Balica S, Popescu I, Jonoski A (2012). Climate change impact on flood hazard, vulnerability and risk of the Long Xuyen Quadrangle in the Mekong Delta. Int. J. River Basin Manag..

[86] Thieler, E. R. & Hammar-Klose, E. S. National assessment of coastal vulnerability to sea-level rise, U.S. Atlantic Coast. US Geological Survey, Open-File Report, 99–593 (1999).

[87] Gornitz V (1991). Global coastal hazards from future sea level rise. Palaeogeogr. Palaeoclimatol. Palaeoecol..

[88] Koroglua A, Ranasinghe R, Iméneze JA, Dastghei A (2019). Comparison of coastal vulnerability index applications for Barcelona province. Ocean Coast. Manag..

[89] Gornitz VM, Daniels RC, White TW, Birdwell KR (1994). The development of a coastal risk assessment database: Vulnerability to sea-level rise in the U.S. southeast. J. Coast. Res..

[90] Szlafsztein C, Sterr H (2007). A GIS-based vulnerability assessment of coastal natural hazards, state of Pará, Brazil. J. Coast. Conserv..

[91] Denner K, Phillips M, Jenkins R, Thomas T (2015). A coastal vulnerability and environmental risk assessment of Loughor Estuary, South Wales. Ocean Coast. Manag..

[92] Carrere, L., Lyard, F. H., Cancet, M. & Guillot, A. Finite element solution fes2014, a new tidal model - validation results and perspectives for improvements. In *ESA Living Planet Conference 2016* (2016).

[93] Adono T (2016). Generation of the 30 M-MESH global digital surface model by ALOS prism. Int. Arch. Photogramm. Remote Sens. Spat. Inf. Sci.- ISPRS Arch..

[94] Zhang K (2019). Accuracy assessment of ASTER, SRTM, ALOS, and TDX DEMs for Hispaniola and implications for mapping vulnerability to coastal flooding. Remote Sens. Environ..

