# Supplementary material for: Coastal vulnerability assessment of the West African coast to flooding and erosion
Source: Sci Rep. 2024 Jan 9;14:890. doi: 10.1038/s41598-023-48612-5 (PMC10776606; doi:10.1038/s41598-023-48612-5)
Supplement: Supplementary file 1 — Supplementary Information. [file 41598_2023_48612_MOESM1_ESM.docx]

**Coastal Vulnerability Assessment of the West African Coast to Flooding and Erosion**

Olusegun A. Dada^1,2*^, Rafael Almar^1*^, Pierre Morand^3^

^1^LEGOS (IRD/CNRS/ CNES/Toulouse University), Toulouse, France; ^2^Dept of Marine Science & Tech., Federal University of Technology Akure, Akure, Nigeria; ^3^UMI SOURCE (IRD - UVSQ/PARIS SACLAY), Guyancourt, France.

*Corresponding Author: oadada@futa.edu.ng

**Supplementary Information**

**Supplementary Results**

**Geophysical variables.** As shown in Figure 4 and Supplementary Figures 1-6, the vulnerability class due to tidal effect shows that 11, 10, 10, 36, and 33 percent of the West African coastline, respectively, have very high, high, moderate, low, and very low vulnerabilities. According to the wave energy ranking category, 17, 22, 24, 18, and 19 percent of the study sites are in the very high, high, moderate, low, and very low vulnerability categories. From the impact of sea level rise, 16, 20, 22, 22 and 20 percent of the study sites have very high, high, moderate, low, and very low vulnerabilities. The classification due to geomorphology indicates that 76 percent of the coastline is very highly vulnerable, while 24 percent is highly vulnerable. The vulnerability class due to coastal slope indicates that 29 percent of the cells are very highly vulnerable, 24 percent are highly vulnerable, 24 percent are moderately vulnerable, 15 percent are at a low level of vulnerability, and 8 percent are at a very low vulnerability level. The classification due to historical shoreline change characteristics indicates 29 percent of the shoreline in grid cells is at very high vulnerability, while 21, 31, 1 and 18 percent of the shoreline, respectively, have a high, moderate, low and very low erosion vulnerability (Fig. 4).

Factors influencing WA (geo)physical processes

**Coastal geomorphology and slope.** Coastal geomorphology is the main dominant variable (Fig. 4c), and it plays a key role here, in those different types of geomorphologies exhibit different categories of vulnerability. The WA coast is comprised of sandy beaches from Mauritania to southern Senegal followed by a muddy form of sedimentation in Guinea and Guinea-Bissau to the north and a sandy form in Liberia and the rest of the WA coast to the south. This large-scale variation reflects a fundamental change in coastal geomorphology and orientation whose implications are expressed as changes in shelf characteristics and coastal lithology (^1^Dada et al., 2021). The WA coastal geomorphology is characterized by deltaic, sandy beaches, and estuarine landforms (^2^Anthony 2006; ^3^Almar et al., 2015). These characteristics give it a “high” to “very high” ranking (Supplementary Fig. 1) and make most areas along the WA coast very susceptible to erosion and flooding.

The coastal slope is low (high risk) for much of the WA coast but becomes higher (lower risk) in some locations (Supplementary Fig. 2). The areas that are most vulnerable to sea-level rise (Fig. 1) have the lowest regional coastal slopes, and geomorphologic types that are susceptible to inundation, wave energy, and/ or the highest rates of shoreline change (Fig. 4). These areas are mostly found along Mauritania, Senegal, Guinea Bissau, and Nigerian Niger Delta coasts (Supplementary Figs 1-6).

For instance, between the central Sierra Leone coast to the Guinea-Bissau coast is a mud-dominated, low wave-energy, meso-macro tidal coast that is distinctly different from the sandy, microtidal, wave-dominated coasts characterizing the rest of the West African coast (^2^Anthony, 2006), excluding the Nigerian Transgressive mud coast (^4,5^Dada et al., 2019, 2020). The morphology of this mud-dominated coast shows variations alongshore that reflect locations of open estuary mouths alternating with muddy intertidal, largely mangrove-colonized plains sometimes comprising cheniers, with bedrock or soft-rock headlands. Its development reflects primary geological heritage involving a wide shallow, low-gradient shelf favourable to the dissipation of waves and tidal amplification (^2^Anthony, 2006).

Further, the vast retreat along the Cote d'Ivoire coastline, according to ^6^Jallow et al. (1999), is caused by the coastal zone being dominated by lowland marshes and lagoons. Coastal retreat along the Gambia coast varies from low retreat in the steeper cliffed zone between Cape St. Mary and Fajara to high retreat in the gently sloping, sandy strand plain around Sanyang Point. ^7^Tano et al. (2016) observed that the western Ivoirian coast is rocky and naturally less exposed to erosion and inundation, therefore it is categorized as in the low-risk category. While they described the Ivoirian coast's central as moderately vulnerable, the eastern coast's high vulnerability is linked to low coastal elevation (^7^Tano et al., 2016).

**Wave climate and associated longshore drift.** The WA coast is exposed to a wave climate characterized by energetic swell and locally generated short-crested waves (^8^Davies, 1980; ^9^Degbe et al., 2010; ^10^Yao et al., 2010), and wave-driven sediment transports are significant drivers of short-term to medium-term shoreline change (^3^Almar et al., 2015; ^11^Anthony et al., 2019). The WA longshore sediment transport (or littoral drift) is one of the largest in the world, variably estimated at between 400,000 and 1,000,000 m^3^/yr and directed eastward (^12^Tastet et al., 1985; ^13^Blivi, 1993; ^14^Anthony and Blivi, 1999). The morpho-sedimentary evolution of the WA coast is controlled by strong longshore sediment transport, resulting in a so-called drift-aligned’ coastline (^15^Anthony, 1995; ^14^Anthony and Blivi, 1999; ^16^Laibi et al., 2014; ^17^Anthony, 2015).

In the Gulf of Guinea, large stretches of the coast are characterized by linear sand barriers built by energetic longshore drift following long and regular sea-swell waves (^3^Almar et al. 2015, 2019; ^17^Anthony 2015; ^11,18^Anthony et al. 2016, 2019; ^19^Ndour et al. 2017). The coastal longshore drift is developed into a single-celled system with the major source of sand from the Volta Delta in Ghana. This coastal area particularly in the Bight of Benin was stable. However, this stability is perturbed by mostly human activities such as the construction of deepwater ports and dams (^3^Almar et al. 2015; ^11^Anthony et al. 2019; ^20^Aman et al. 2019), thereby causing local erosion (Rossi, 1989). Wave actions combined with human activities will make most of the WA coastal areas more vulnerable (^21^IPCC, 2021; ^1^Dada et al., 2021; ^22^Cisse et al., 2022; ^23^Sakho et al., 2022). A recent study of the past and future events of the extremes of wave conditions in the Gulf of Guinea (GoG) shows that the average values of annual and seasonal significant wave heights and wave periods would increase by 0.1 m and 0.29 s, respectively, by the end of the century. The mean wave direction is projected to become more southwesterly with an increase of up to 2.2° by 2100 (^24^Dahunsi et al., 2022).

**Shoreline change.** The sub-Saharan African coast accounts for 148,000 km^2^ of low-elevation coastal zones, with an exposed population of around 45 million people (^25^Neumann et al., 2015). A large part of the West African coastal zone is composed of sedimentary, sandy, naturally mobile, and dynamic shorelines. The dynamics of these mobile shorelines are conditioned by the circulation of sediment carried by a coastal drift parallel to the coast (^26^UEMOA/MOLOA, 2017). Coastal evolution and shoreline trends, such as erosion, are associated with physical (coastal) process interactions and sediment linkages between different coastal landform units. Patterns of WA shoreline change significantly, depending on the geomorphic setting, sediment availability, and wave/ sediment cell dynamics. The WA river regime is marked by heavy discharge and sediment supply to the coastal zone in the wet season months (^2^Anthony, 2006). Some of these shorelines have been experiencing erosion resulting from alongshore port construction and reduced sediment supply due to river damming (^11^Anthony et al. 2019; ^16^Laibi et al., 2014; ^3,27^Almar et al. 2015, 2019; ^28^Giardino et al. 2017). For example, southern Nouakchott is experiencing strong erosion due to the disruption of the coastal drift by developments of the port while the entire Volta delta coast and downstream adjacent areas in Togo and Benin are eroding owing to the damming of the upper Volta River (^11^Anthony et al. 2019; ^16^Laibi et al 2014; ^3,27^Almar et al. 2015, 2019; ^28^Giardino et al. 2017). These deltas are equally gradually sinking under the sediment load due to irregular supply with new sediment inputs.

**Supplementary Table**

Table 1. Quantitative and qualitative ranking ranges of vulnerability variables for the WA African coast.

| Indicator | Source | Symbol | Index Value | | | | |
| --- | --- | --- | --- | --- | --- | --- | --- |
|  |  |  | **Very Low (1)** | **Low (2)** | **Moderate (3)** | **High (4)** | **Very High (5)** |
| Physical Vulnerability Variables (PVI) | | | | | | | |
| Geomorphology | Satellite Imagery, Google Earth | GEO | Rocky/ Hard Cliffs | Medium cliffs indented coasts | Low cliffs, Glacial drift, Alluvial plains | Cobble beaches, Estuary Lagoon | Barrier beaches, Sand beaches, Salt marsh, Mudflats, Deltas, Mangroves, Coral reefs |
| Coastal slopes (%) | Satellite Imagery, Google Earth | CS | >1 | 1 to 0.5 | 0.5 to 0.1 | 0.1 to 0.05 | <0.05 |
| Shoreline change rate (m) | Satellite Imagery | SCR | >+2 | 1 to 2 | 1 to -1 | -1 to -2 | <-2 |
| Sea Level rise (mm/yr) | Satellite altimetry | SLC | <-1.21 | -1.21 to 0.1 | 0.1 to 1.24 | 1.24 to 1.36 | <1.36 |
| Wave energy (Wm−1) | Modeled | WE | <1.1 | 1.1 to 2.0 | 2.1 to 2.25 | 2.25 to 2.60 | >2.70 |
| Tidal range (m) | Modeled | TR | >6.0 | 4.1 to 6.0 | 4.0 to 2.0 | 1.0 to 1.9 | <1.0 |
| Socioeconomic Vulnerability Variables (SVI) | | | | | | | |
| Population density | Map | POP | <10 | 10 to 50 | 50 to 100 | 100 to 500 | > 500 |
| Human settlement (population growth, built-up environments, and urbanization degree) | Map | SETT | <10 | 10 to 30 | 30 to 50 | 50 to 75 | > 75 |

**Supplementary Figures**


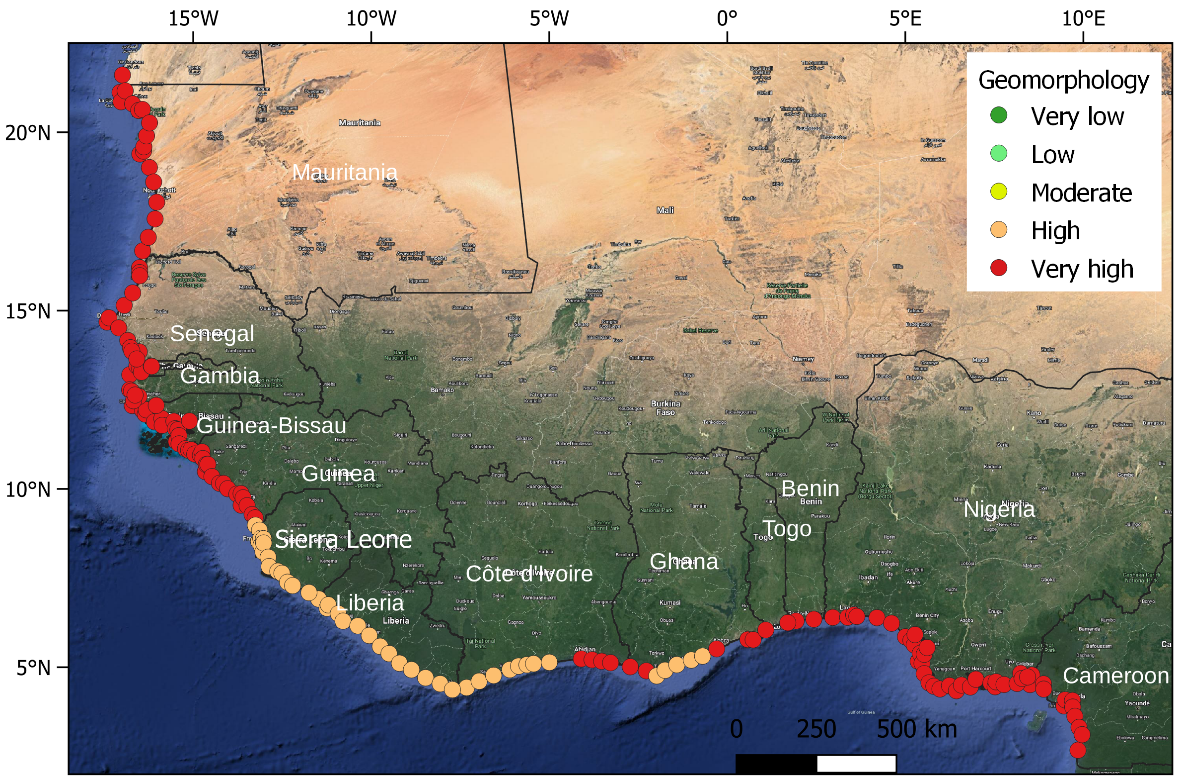


Supplementary Figure 1. Coastal geomorphology of West Africa. The various coastal geomorphology along the coast is depicted by the coloured coastline. Barrier islands, salt marshes, tidal flats, deltas, and sand beaches are all parts of the very high-vulnerability geomorphology (red), whereas estuaries and lagoons are part of the high-vulnerability geomorphology (orange). (The map image used in producing the figure was generated using the Google Satellite Hybrid plugin in QGIS v.3.24.0 environment, [<https://www.qgis.org/>](https://www.qgis.org/)).


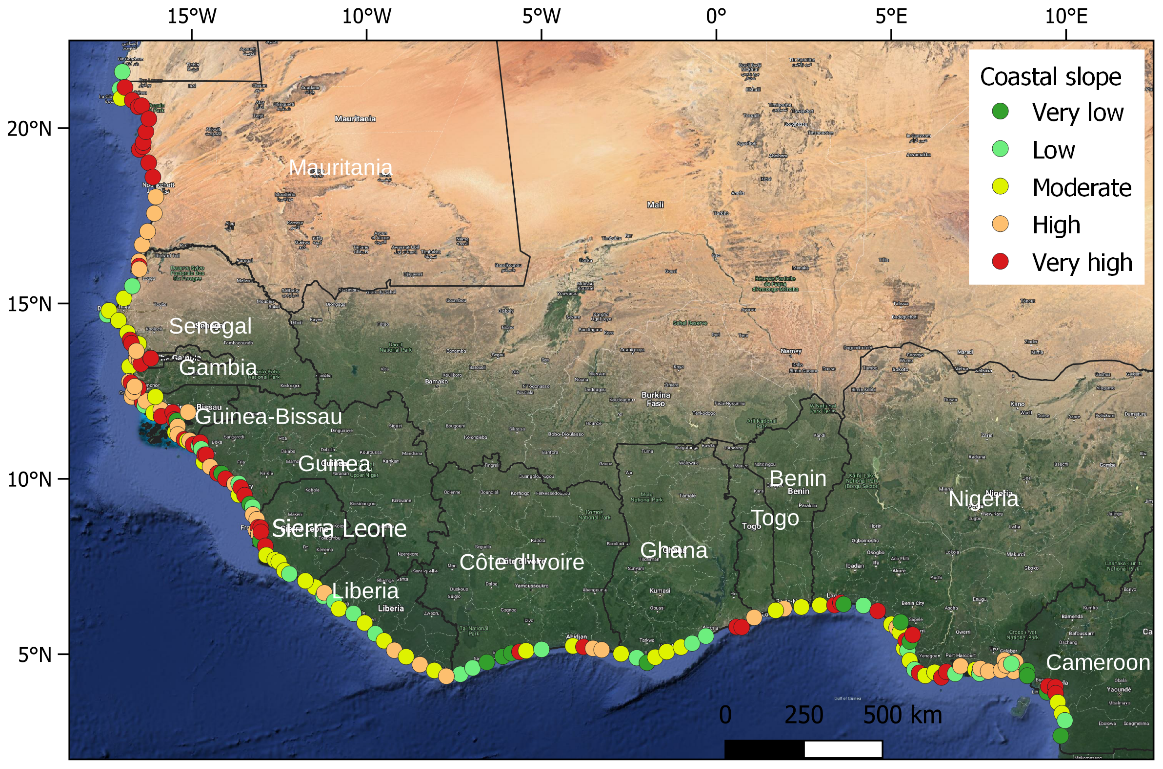


Supplementary Figure 2. The regional coastal slope for West Africa. The coloured shoreline represents the regional slope of the coast. Very high vulnerability slope areas (in red) are the gentlest regarding the CVI ranking system. (The map image used in producing the figure was generated using the Google Satellite Hybrid plugin in QGIS v.3.24.0 environment, [<https://www.qgis.org/>](https://www.qgis.org/)).


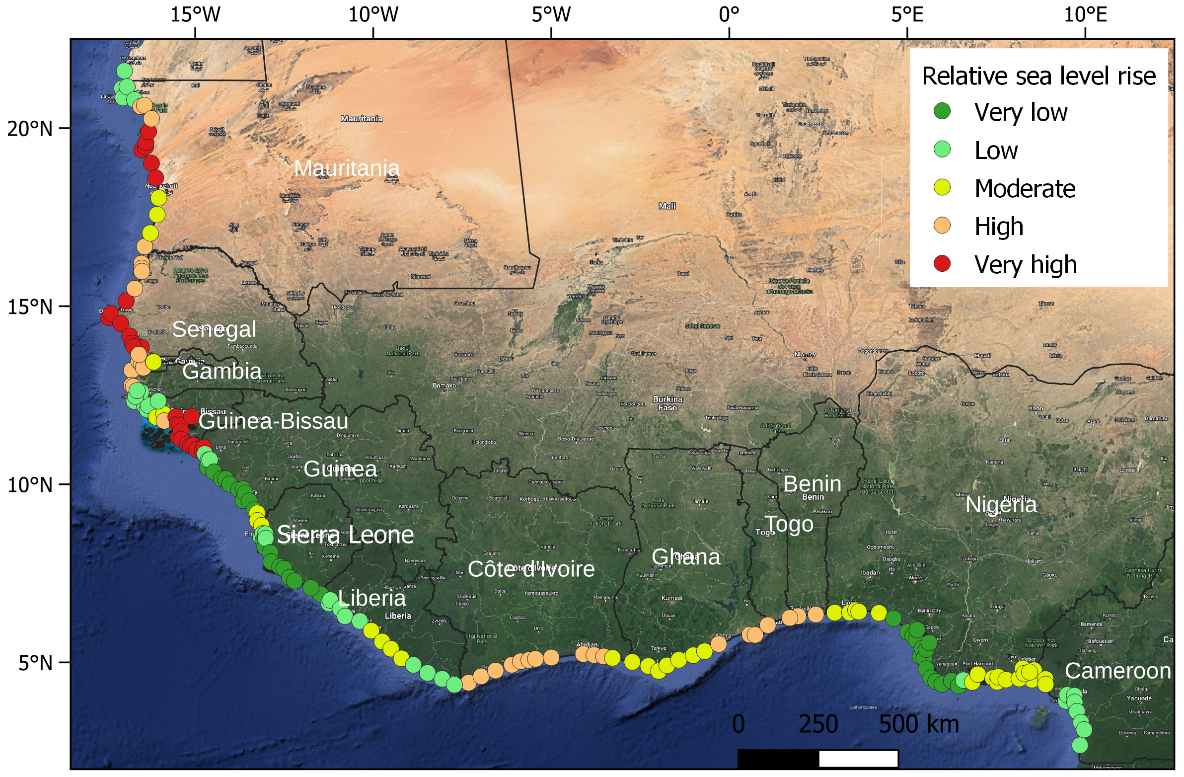


Supplementary Figure 3. Relative sea-level change rate along the WA coast. (The map image used in producing the figure was generated using the Google Satellite Hybrid plugin in QGIS v.3.24.0 environment, [<https://www.qgis.org/>](https://www.qgis.org/)).


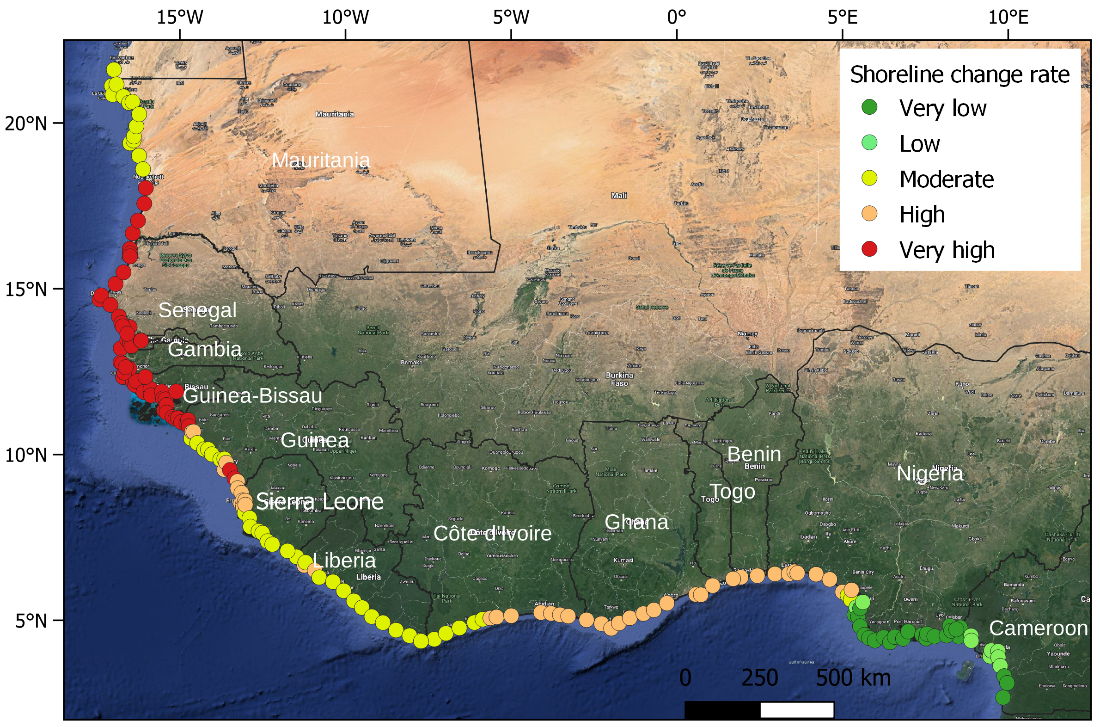


Supplementary Figure 4. Historical shorelines change rate along the WA coast. (The map image used in producing the figure was generated using the Google Satellite Hybrid plugin in QGIS v.3.24.0 environment, [<https://www.qgis.org/>](https://www.qgis.org/)).


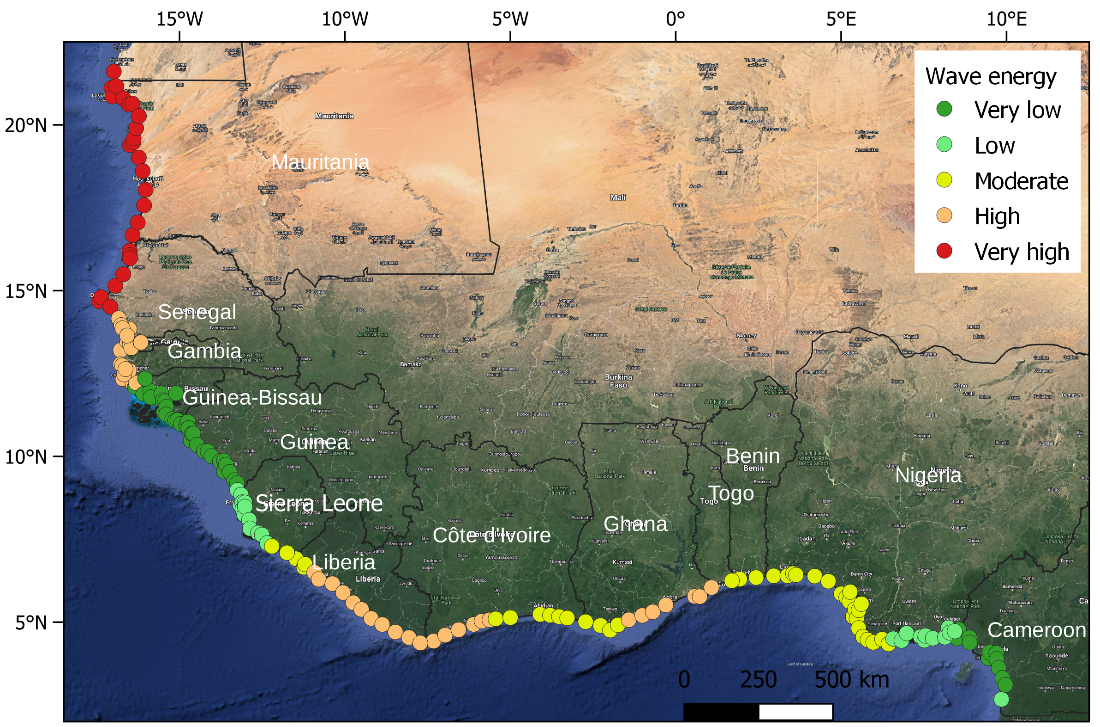


Supplementary Figure 5. Wave energy ranking along the WA coast. (The map image used in producing the figure was generated using the Google Satellite Hybrid plugin in QGIS v.3.24.0 environment, [<https://www.qgis.org/>](https://www.qgis.org/)).


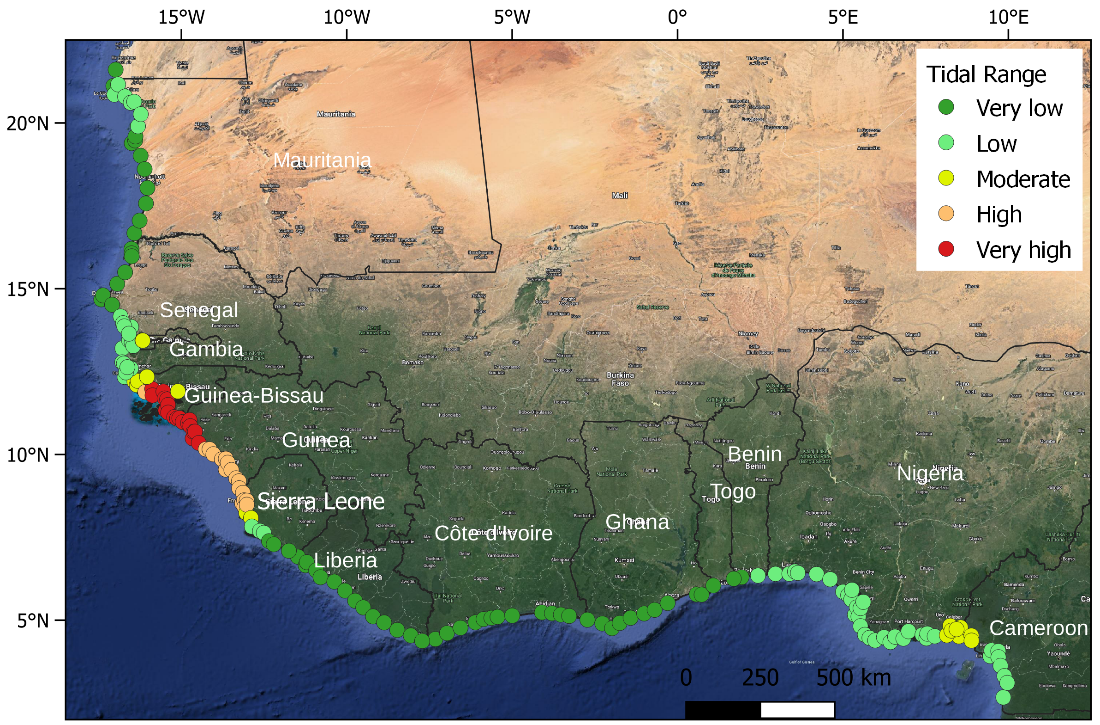


Supplementary Figure 6. Tidal range ranking along the WA coast. (The map image used in producing the figure was generated using the Google Satellite Hybrid plugin in QGIS v.3.24.0 environment, [<https://www.qgis.org/>](https://www.qgis.org/)).


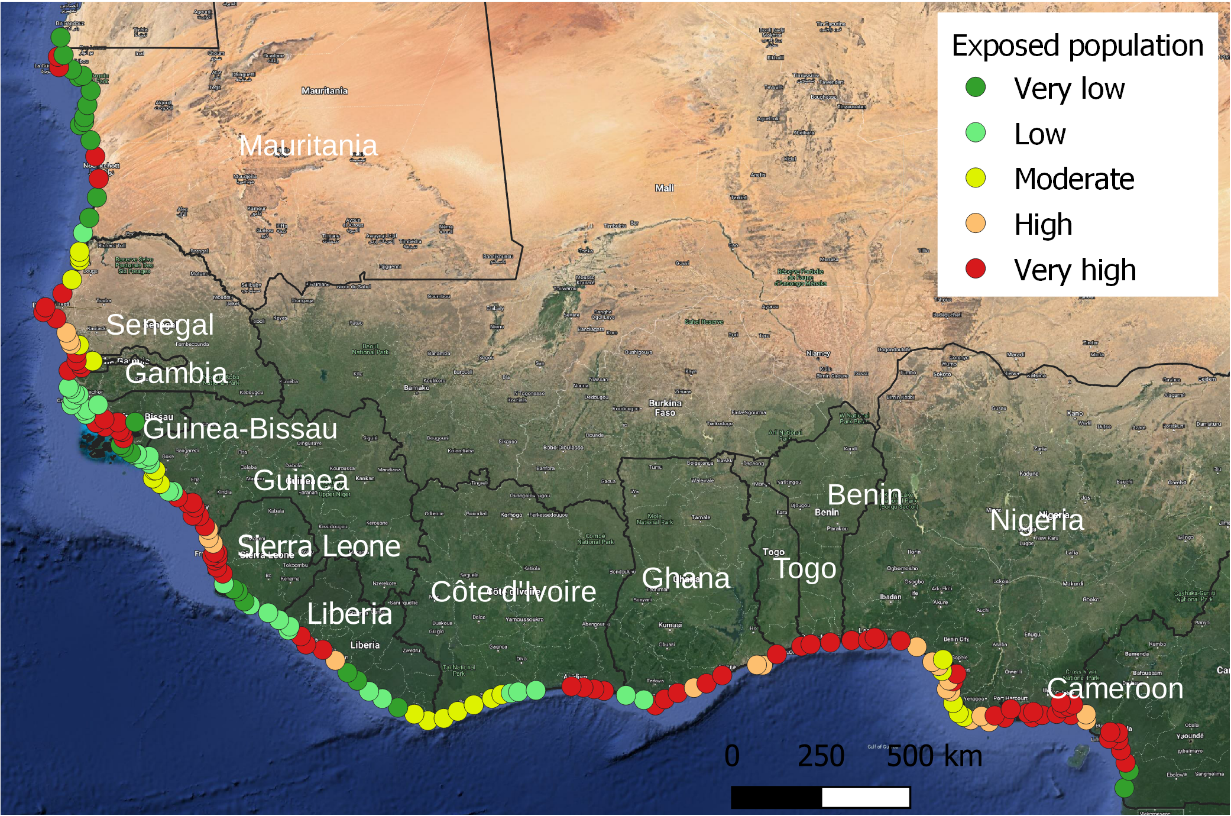


Supplementary Figure 7. West Africa exposed population. (The map image used in producing the figure was generated using the Google Satellite Hybrid plugin in QGIS v.3.24.0 environment, [<https://www.qgis.org/>](https://www.qgis.org/)).


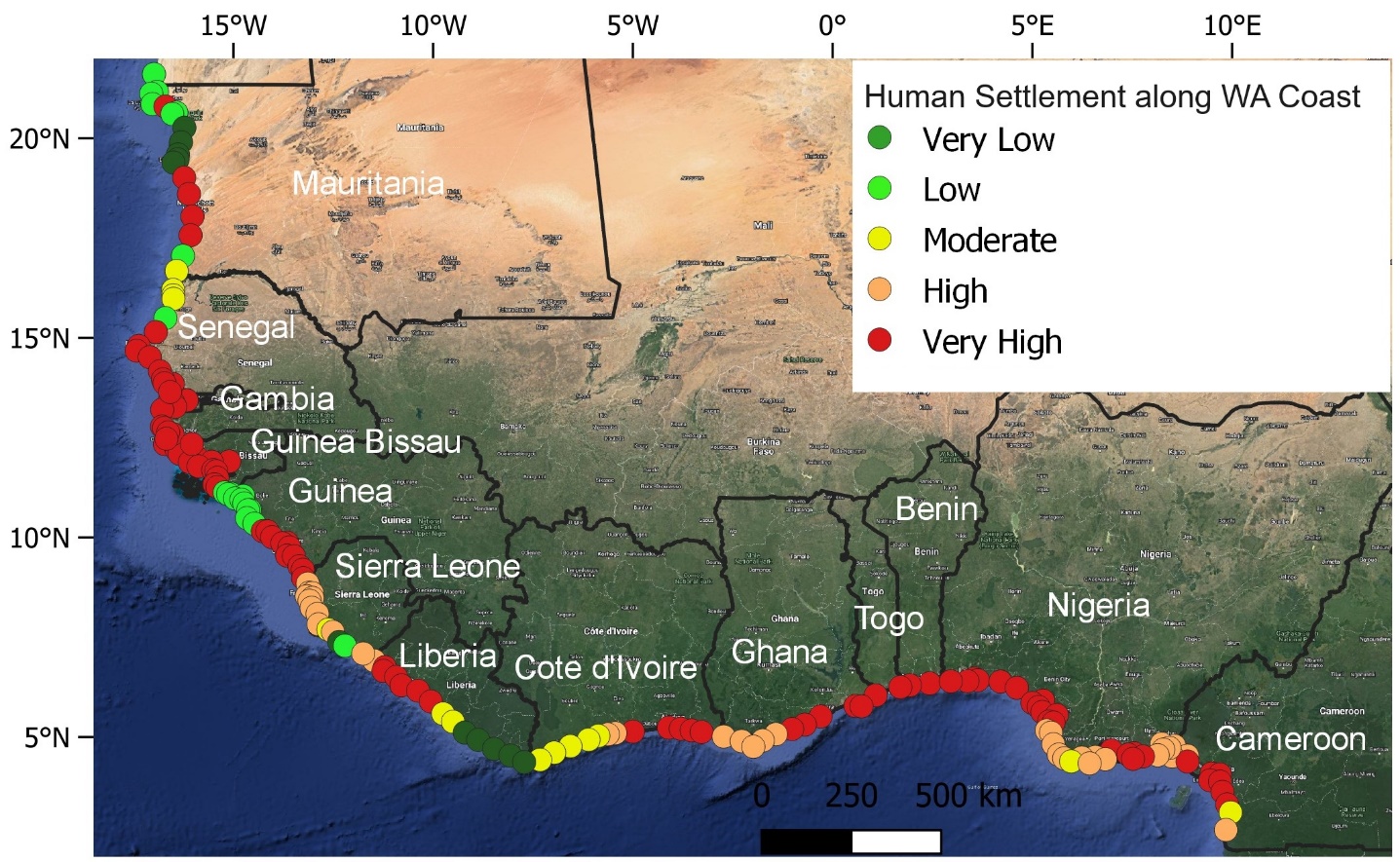


Supplementary Figure 8. West Africa human settlement. (The map image used in producing the figure was generated using the Google Satellite Hybrid plugin in QGIS v.3.24.0 environment, [<https://www.qgis.org/>](https://www.qgis.org/)).

Supplementary References

1. Dada, O., Almar, R., Morand, P. & Menard, F. Towards West African coastal social-ecosystems sustainability: Interdisciplinary approaches. Ocean and Coastal Management 211, 105746 (2021).

2. Anthony EJ. The muddy tropical coast of West Africa from Sierra Leone to Guinea-Bissau: geological heritage, geomorphology and sediment dynamics. Africa Geoscience Review13:227–237 (2006).

3. Almar R, Kestenare E, Reyns J, Jouanno J, Anthony EJ, Laïbi R, Hemer M, Du Penhoat Y, Ranasinghe R. Response of the bight of Benin (gulf of Guinea, West Africa) coastline to anthropogenic and natural forcing, part 1: wave climate variability and impacts on the longshore sediment transport. Cont Shelf Res 110:48–59 (2015).

5. Dada, O.A., Almar, R. & Oladapo, M.I. Recent coastal sea-level variations and flooding events in the Nigerian Transgressive Mud coast of Gulf of Guinea. J. African Earth Sciences 161, 103668 (2020).

6. Jallow, B.P., Toure, S., Barrow, M.M.K. and Mathieu, A.A. Coastal zone of The Gambia and the Abidjan region in Côte d'Ivoire: sea level rise vulnerability, response strategies, and adaptation options. Climate Research 12 (2/3), CR Special 6: National assessment results of climate change: impacts and responses 129-136 (1999).

7. Tano, R.A., Aman, A., Kouadio, K.Y., Toualy, E., Ali, K.E. and Assamoi, P. Assessment of the Ivorian Coastal Vulnerability. Journal of Coastal Research, 32, 1495- 1503 (2016).

8. Davies, J.L. Geographical Variation in Coastal Development. 2nd Ed. Longman, London, 212 pp (1980).

9. Degbe C.G., OyedeL.M., Laibi, R.. Risques environnementaux sur le littoral béninois:

érosion côtière et stratégies de lutte. Journées Internationales du Dragage, Libreville, Unpub. (2010).

10. Yao, K.S., Abe, J., Bamba, S.B., Konan, K.E., and Aka, K.. Dynamique d’un perim ´ etre littoral portuaire: La c ` ote de San-P ˆ edro, ´ Sud-Ouest de la Cote d’Ivoire. ˆ Revue Paralia, 3, 2.1–2.12 (2010).

11. Anthony EJ, Almar R, Besset M, Reyns J, Laïbi R, Ranasinghe R, Abessolo Ondoa G, Vacchi M Response of the bight of Benin (Gulf of Guinea, West Africa) coastline to anthropogenic and natural forcing, part 2: sources and patterns of sediment supply, sediment cells, andrecent shorelinechange. Cont ShelfRes173:93– 103 (2019).

12. Tastet J.P., Caillon L., Simon B. La dynamique sédimentaire littorale devant Abidjan : Impact des aménagements. Contribution à la compréhension des phénomènes d’érosion et de sédimentation. Université d’Abidjan (FAST), PAA, 46p (1985).

13. Blivi, A. Morphology and current dynamic of the coast of Togo. In: Coastlines of Western Africa. Géo-Eco-Trop. 17 (1-4): 21-35 (1993).

14. Anthony EJ. & Blivi A. Morphosedimentary evolution of a deltasourced, drift-aligned sand barrier-lagoon complex, western bight of Benin. Mar Geol 158:161–176 (1999).
15. Anthony, E. J. (1995). Beach-ridge development and sediment supply: Examples from West Africa. *Marine Geology*, *129*(1-2), 175-186. https://doi.org/10.1016/0025-3227(95)00111-5
16. Laïbi, RA, Anthony, EJ, Almar, R, Castelle, B, Sénéchal, N, Kestenare, E. Longshore drift cell development on the human-impacted Bight of Benin sand barrier coast, West Africa. J Coast Res 70(SI): 78–83 (2014).

17. Anthony, E.J. Patterns of sand spit development and their management implications on deltaic, drift-aligned coasts: the cases of the Senegal and Volta River delta spits, West Africa. In: Randazzo, G., Cooper, J.A.G. (ed), Sand and Gravel Spits. Coastal Research Library Series 12, Springer, pp. 21–36 (2015).
18. Anthony EJ, Almar R, Aagaard T. Recent shoreline changes in the Volta River delta, West Africa: the roles of natural processes and human impacts. Afr J Aquat Sci 41:81–87 (2016).

19. Ndour, A., Laïbi, R.A., Sadio, M., Degbe, C., Degbe, E., Diaw, A.T., Oyede, L., Anthony, E.J., Dussouillez, P., Sambou, H., Dieye, E.B. Management strategies for coastal erosion problems in WestAfrica: analysis, issues, and constraints drawn fromthe examples of Senegal and Benin. Ocean Coast Manage 156:92–106 (2017).

20. Aman A, Tano RA, Toualy E, Silué F, Appeaning Addo K, & Folorunsho R. Physical forcing induced coastal vulnerability along the Gulf of Guinea. J Environ Prot 10:1194–1211 (2019).

21. IPCC. Climate Change 2021: The Physical Science Basis. Contribution of Working Group I to the Sixth Assessment Report of the Intergovernmental Panel on Climate Change (2021).

22. Cisse, C. O., Brempong, E. K., Taveneau, A., Almar, R., Sy, B. A., & Angnuureng, D. B. Extreme coastal water levels with potential flooding risk at the low-lying Saint Louis historic city, Senegal (West Africa). *Frontiers in Marine Science*, *9*, 993644 (2022).

23. Sakho, I., Sadio, M., Camara, I., Noblet, M., Seck, A., Saengsupavanich, C., Ndour, A., Diouf, M.B. Sea level rise and future shoreline changes along the sandy coast of Saloum Delta, Senegal. Arabian J. Geosci. 15 (19), 1547 (2022).
24. Dahunsi, A.M., Bonou, F., Dada, O.A., Baloïtcha, E. Spatio-Temporal Trend of Past and Future Extreme Wave Climates in the Gulf of Guinea Driven by Climate Change. J. Mar. Sci. Eng. 10, 1581 (2022).

25. Neumann, B., Vafeidis, A.T., Zimmermann, J., Nicholls, R.J.B. Future coastal population growth and exposure to sea-level rise and coastal flooding—A global assessment. *PLoS ONE*  *10*, e0118571 (2015).

26. UEMOA/MOLOA. Assessment 2016 West Africa Coastal Areas General Document, 148p (2017).

27. Almar, R., Kestenare, E. and Boucharel, J. On the key influence of remote climate variability from Tropical Cyclones, North and South Atlantic mid-latitude storms on the Senegalese coast (West Africa). Environ. Res. Commun. 1, 071001 (2019).

28. Giardino, A., Schrijvershof, R., Nederhoff, C.M., de Vroeg, H., Brière, C., Tonnon, P.K., Caires, S., Walstra, D.J., Sosa, J., van Verseveld, W., Schellekens, J., Sloff, C.J. A quantitative assessment of human interventions and climate change on the west African sediment budget. Ocean Coast Manag 156:249–265 (2018).
